# Multi-tiered approach to detect autoimmune cross-reactivity of therapeutic T cell receptors

**DOI:** 10.1126/sciadv.adg9845

**Published:** 2023-07-26

**Authors:** Kazusa Ishii, John S. Davies, Andrew L. Sinkoe, Kilyna A. Nguyen, Scott M. Norberg, Crystal P. McIntosh, Tejas Kadakia, Carylinda Serna, Zachary Rae, Michael C. Kelly, Christian S. Hinrichs

**Affiliations:** ^1^ Center for Immuno-Oncology, Center for Cancer Research (CCR), National Cancer Institute (NCI), National Institutes of Health (NIH), Bethesda, MD, USA.; ^2^ Department of Safety Assessment, Genentech Inc., South San Francisco, CA, USA.; ^3^ Precigen, Germantown, MD, USA.; ^4^ Oncology Department, Cell Therapy Unit, AstraZeneca, Gaithersburg, MD, USA.; ^5^ Single Cell Analysis Facility, CCR, NCI, NIH, Bethesda, MD, USA.; ^6^ 10x Genomics, Pleasanton, CA, USA.; ^7^ Duncan and Nancy MacMillan Center of Excellence in Cancer Immunotherapy and Metabolism, Rutgers Cancer Institute of New Jersey, New Brunswick, NJ, USA.

## Abstract

T cell receptor (TCR)–engineered T cell therapy using high-affinity TCRs is a promising treatment modality for cancer. Discovery of high-affinity TCRs especially against self-antigens can require approaches that circumvent central tolerance, which may increase the risk of cross-reactivity. Despite the potential for toxicity, no standardized approach to screen cross-reactivity has been established in the context of preclinical safety evaluation. Here, we describe a practical framework to prospectively detect clinically prohibitive cross-reactivity of therapeutic TCR candidates. Cross-reactivity screening consisted of multifaceted series of assays including assessment of p-MHC tetramer binding, cell line recognition, and reactivity against candidate peptide libraries. Peptide libraries were generated using conventional contact residue motif–guided search, amino acid substitution matrix–based search unguided by motif information, and combinatorial peptide library scan–guided search. We demonstrate the additive nature of a layered approach, which efficiently identifies unsafe cross-reactivity including one undetected by conventional motif-guided search. These findings have important implications for the safe development of TCR-based therapies.

## Teaser

Multitiered approach identifies unsafe TCR cross-reactivity including those undetected by conventional motif-guided screening.

## INTRODUCTION

Adoptive transfer of T cell receptor (TCR)–engineered T cells has emerged as a promising anticancer strategy. In addition to neoantigens derived from cancer-specific mutations (*1*) and viral gene products (*2*, *3*), self-antigens can be exploited as targets for TCR-engineered T cell therapy when their expression is limited to cancers and dispensable normal tissues such as B cells. Clinical studies of adoptive transfer of self-antigen–directed T cells, manufactured either by ex vivo expansion of peripheral blood mononuclear cells (PBMCs) with self-antigen–derived peptides (*4*, *5*) or by TCR engineering (*6*–*8*), have demonstrated favorable outcomes, supporting that TCRs targeting select self-antigens may play an important role in cancer regression. Furthermore, tumor-infiltrating lymphocytes have been shown to contain self-antigen–specific TCRs likely responsible for mediating clinical responses (*9*).

One of the main safety concerns for TCR-engineered T cell therapies using high-affinity TCRs is off-target toxicity (cross-reactivity) (*10*–*12*). High-affinity TCRs against self-antigens are usually deleted through thymic selection processes. Therefore, discovery of high-affinity TCRs against self-antigens can require approaches that circumvent central tolerance, such as interrogation of TCR repertoire of mice or allogeneic donors (*13*). Moreover, TCR affinity enhancement processes are often used to increase the TCR affinity for its ligand to the level necessary to achieve robust anticancer responses (*14*, *15*). However, TCRs not subject to thymic selection pose a greater risk of mediating cross-reactivity against unintended targets. Historically, severe off-target clinical toxicities have been reported only in affinity-enhanced TCRs against self-antigens (*10*–*12*). These examples signify the difficulties of predicting clinically consequential cross-reactivity of TCRs that bypass central tolerance.

Despite the potential clinical consequence, there is currently no standardized approach to efficiently screen for cross-reactivity in the context of preclinical safety evaluation. There are no animal models, tissue arrays, or cell lines that adequately cover the whole scope of the human proteome and human leukocyte antigen (HLA) diversity. Therefore, off-target toxicities of TCR-engineered T cell therapy are generally evaluated by screening the TCR against a panel or library of peptide-HLA complexes, such as cell lines loaded with candidate peptides. A widely used method to curate candidate peptides for cross-reactivity screening involves identification of TCR/peptide–major histocompatibility complex (p-MHC) contact residues using amino acid scanning approaches followed by in silico search of peptides sharing the contact residue motifs (*2*, *10*). However, there are examples of TCRs in experimental settings that required far more comprehensive approaches for epitope detection, such as use of a combinatorial peptide library (CPL) (*16*–*18*) or yeast library (*19*–*21*). A streamlined approach to screen for cross-reactivity is necessary to promptly and safely bring a TCR to the clinic, especially when the candidate TCR is not from an autologous TCR repertoire.

This study aimed to establish a practical approach to screen for cross-reactivity of therapeutic TCRs. Using a panel of newly isolated TCRs that recognize an HLA-A*02:01–restricted epitope of human CD20, we first show that the most cross-reactive TCRs could be rapidly detected with p-MHC tetramer screening and small coculture screening assays. For the TCR that demonstrated target recognition pattern restricted by the intended antigen and HLA, target peptide residues critical for TCR engagement (recognition motif or contact motif) were identified using alanine substitution assay, which informed in silico search for peptides from human proteome that shared the TCR recognition motif. In addition, in silico search was broadened to peptides with substitution of residues with physically and biochemically similar amino acids, unguided by recognition motif information. Using this systematic approach, clinically prohibitive TCR cross-reactivity could be successfully detected even when the cross-reactive peptide did not have the TCR recognition motif of the intended epitope. Different in silico search rules, including the extensive CPL-based search, were compared for their efficiency and ability to detect the cross-reactive peptide. Applicability of the approach to other TCRs were evaluated in historical TCRs with known cross-reactivity.

## RESULTS

### Rapid screening for cross-reactivity using p-MHC tetramer staining and coculture assays

**Fig. 1.**
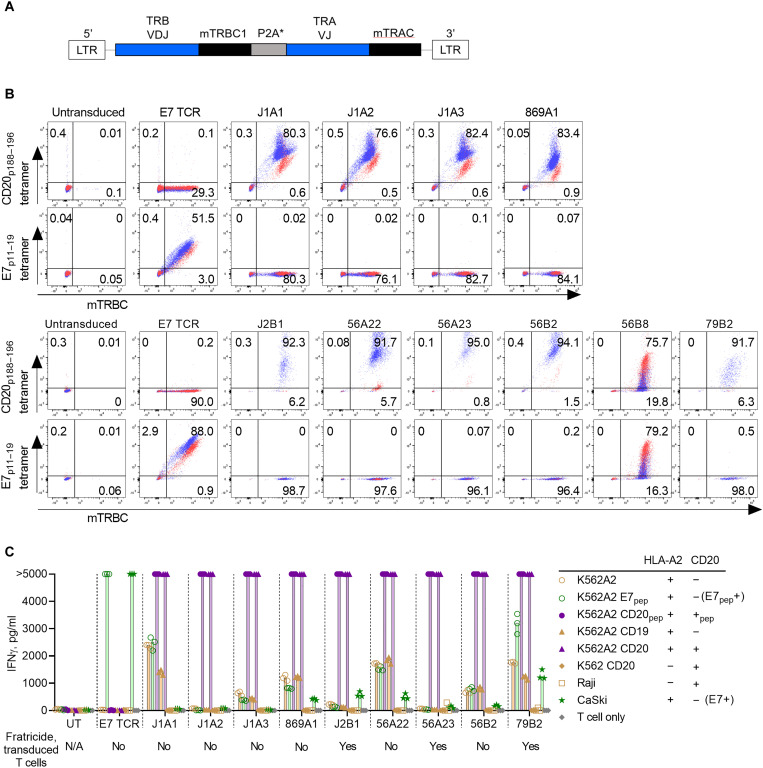
Rapid screening of TCR cross-reactivity using p-MHC tetramer and a coculture assay. (**A**) TCR β and α chains were cloned into a gamma-retroviral vector (MSGV1) in a bicistronic format with a cleavable linker (furin recognition sequence, SGSG linker, and P2A). Murine constant regions were modified as previously described (see Materials and Methods). (**B**) Human T cells were transduced with either E7 TCR or each CD20 TCR clone. Cell surface tetramer binding and murine TCR β constant region (mTRBC) expression were assessed with flow cytometry. CD8^+^ and CD4^+^ T cells are highlighted in blue and red, respectively. (**C**) TCR-transduced T cells were cocultured with target cell lines at an effector-to-target (E:T) ratio of 1:1 (5 × 10^4^ cells each in 96-well U-bottom plate). IFNγ levels of the overnight coculture supernatant were measured with ELISA. Technical replicates: (C) *n* = 3. Representative of three independently performed experiments with independent donor PBMCs. Levels above the detection limit of this assay (5000 pg/ml) are shown as >5000 pg/ml.

A panel of 10 unique murine TCRs that recognize HLA-A*02:01–restricted epitope of human CD20 (CD20_p188–196_, SLFLGILSV) were isolated from HLA-A2 transgenic mice vaccinated with the epitope peptide (fig. S1, table S1, and Supplementary Methods). Each TCR was reconstructed and cloned into a retroviral expression vector (Fig. 1A and Materials and Methods). Human polyclonal T cells were transduced to express each anti-CD20 TCR. First, TCRs were evaluated whether they bound specifically to cognate p-MHC tetramers (Fig. 1B). Of 10 unique TCRs, the clone 56B8 was found to nonspecifically interact with HLA-A*02:01, binding to both HLA-A*02:01–CD20_p188–196_ tetramers (“CD20 tetramers” hereafter) and HLA-A*02:01–E7_p11–19_ tetramers (“E7 tetramers” hereafter) (Fig. 1B). T cells transduced to express each of the rest of nine anti-CD20 TCR clones (anti-CD20 TCR T cells) bound only to CD20 tetramers but not to E7 tetramers. To further characterize the specificity of TCRs, anti-CD20 TCR T cells were cocultured with a panel of cell lines (Fig. 1C). T cells expressing the clone J1A2 (J1A2 T cells) recognized target cell lines only when they expressed both HLA-A*02:01 and CD20 (either full-length CD20 protein or loaded with the CD20_p188–196_ peptide). The remaining eight clones (J1A1, J1A3, 869A1, J2B1, 56A22, 56A23, 56B2, and 79B2) all exhibited possible signs of TCR cross-reactivity characterized by low-grade interferon-γ (IFNγ) production in recognition of HLA-A*02:01^+^ cells without CD20 expression or potential severe fratricides of TCR-transduced T cells when transduced cells expressed HLA-A*02:01 (fig. S2). On the basis of these findings, the clone J1A2 was chosen for further evaluation.

### Evaluation of specificity and functional avidity of J1A2 T cells

**Fig. 2.**
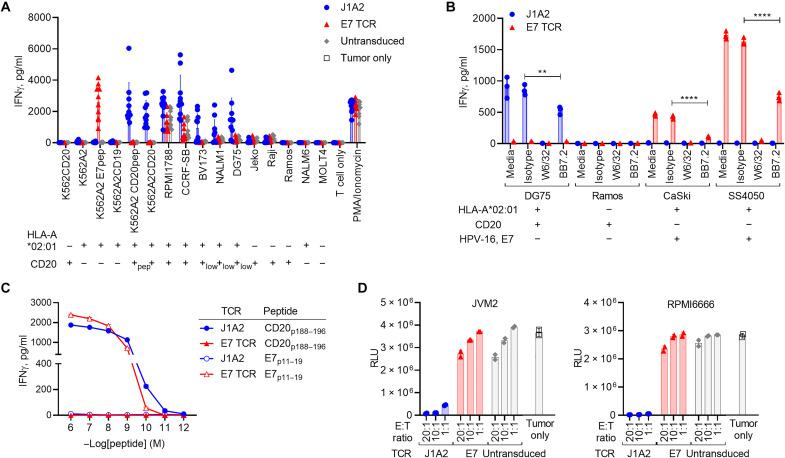
J1A2 TCR–transduced T cells have high functional avidity and specifically kill target cell lines. (**A**) TCR-transduced T cells were cocultured with target cell lines indicated on the *x* axis at an E:T ratio of 1:1 (5 × 10^4^ cells each in 96-well U-bottom plate). IFNγ levels of the overnight coculture supernatant were measured with ELISA. Results from 10 independent donors are shown. (**B**) TCR-transduced T cells were cocultured with target cell lines indicated on the *x* axis at an E:T ratio of 1:1 (5 × 10^4^ cells each in 96-well U-bottom plate) in the presence of 50 μg/ml of either pan–HLA class I antibody (W6/32), anti–HLA-A2 antibody (BB7.2), isotype control, or media alone. IFNγ levels of the overnight coculture supernatant were measured with ELISA. (**C**) Human T cells transduced with either J1A2 TCR or E7 TCR were cocultured with K562A2 loaded with either CD20_p188–196_ or E7_p11–19_ peptides at concentrations indicated on the *x* axis at an E:T ratio of 1:1 (5 × 10^4^ cells each in 96-well U-bottom plate). IFNγ levels of the overnight coculture supernatant were measured with ELISA. (**D**) Bioluminescence-based in vitro cytotoxicity assay: Cell lines RPMI6666 and JVM2 are both HLA-A*02:01^+^ and CD20^+^. J1A2 TCR– or E7 TCR–transduced T cells were cocultured with firefly luciferase–expressing RPMI6666 or JVM2 at E:T ratios indicated in *x* axes. Six hours after coculture, the level of luminescence was measured immediately following the addition of luciferin. RLU, Relative Light Unit. Biological replicates: (A) *n* = 10. Technical replicates: (B) *n* = 3, (C) *n* = 2, and (D) *n* = 2. Representative of (B) and (C) three and (A) and (D) two independently performed experiments with independent donor PBMCs. Statistical significance was determined using ANOVA with Sidak’s correction (B). Data are reported as mean ± SD. ****P < 0.0001 and **P < 0.01.

A series of experiments was performed to further characterize J1A2 T cells. J1A2 T cells were capable of recognizing a large panel of target cell lines, including B cell leukemia, lymphoma, and Epstein-Barr virus-transformed lymphoblastoid cell lines (EBV-LCL), in a manner restricted by both HLA-A*02:01 and CD20 (Fig. 2A and fig. S3). Multiple cell lines that lacked either HLA-A*02:01 or CD20 did not elicit IFNγ secretion by J1A2 T cells, suggesting that J1A2 T cells are not cross-reactive against the set of HLA alleles (data file S5) and immunopeptidome expressed by these cell lines. HLA class I–blocking antibody (clone W6/32) and HLA-A2–blocking antibody (clone BB7.2) either completely or partially blocked IFNγ secretion by J1A2 T cells upon coculturing with a CD20^+^/HLA-A*02:01^+^ target cell line (Fig. 2B). These findings reaffirmed that J1A2 T cells recognize an HLA-A*02:01–restricted epitope of CD20.

J1A2 T cells were able to recognize the K562A2 cell line loaded with cognate peptide at a concentration as low as 10^−10^ M (Fig. 2C), which was equivalent to the lowest concentration of the E7_p11–19_ peptide recognized by the clinically active E7 TCR T cells (*3*). J1A2 T cells were able to mediate in vitro cytotoxicity against HLA-A*02:01^+^ and CD20^+^ mantle cell lymphoma cell line (JVM2) and Hodgkin lymphoma cell line (RPMI6666) (Fig. 2D). Collectively, these data indicate that J1A2 T cells have high functional avidity comparable to another TCR that has demonstrated antitumor activity in patients.

### Conventional TCR recognition motif-guided cross-reactivity screening

**Fig. 3.**
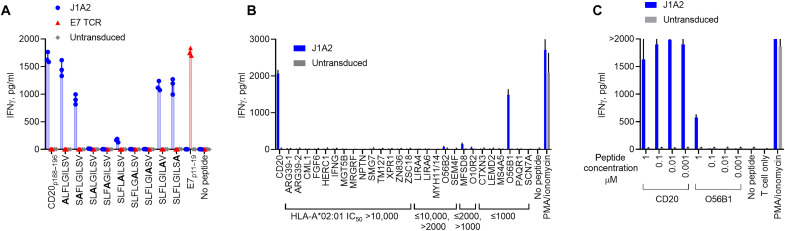
Peptide-TCR contact residue identification and motif-guided cross-reactivity screening. (**A**) TCR-transduced T cells were cocultured with K562A2 loaded with 1 μM of peptides CD20_p188–196_, E7_p11–19_, or with CD20 epitope with alanine substitution at one residue at a time. IFNγ levels of the overnight coculture supernatant were measured with ELISA. (**B**) J1A2 T cells were cocultured with K562A2 loaded with each peptide at 1 μM. Candidate peptides share the TCR recognition motif. IFNγ levels of the overnight coculture supernatant were measured with ELISA. PMA, phorbol 12-myristate 13-acetate. (**C**) J1A2 T cells or untransduced T cells were cocultured with K562A2 cells loaded with either CD20 epitope peptide or the O56B1-derived peptide at titrated concentrations as indicated on the *x* axis. IFNγ levels of the overnight coculture supernatant were measured with ELISA. Technical replicates: (A) *n* = 3, (B) *n* = 2, and (C) *n* = 2. Representative of (A) four and (B) and (C) three independently performed experiments with independent donor PBMCs.

For a TCR to be safely translated into clinic, prohibitive cross-reactivity must be ruled out to avoid unintended target recognition, which could lead to off-target toxicities. The established method of alanine scanning to determine the residues critical for TCR recognition followed by in silico search of peptides sharing the TCR recognition motif was used to evaluate cross-reactivity of J1A2 TCR. This method has previously been shown to effectively identify cross-reactive peptides (*10*, *11*). To determine which amino acid residues in the CD20_p188–196_ peptide were critical for TCR recognition, J1A2 T cells were cocultured with HLA-A*02:01^+^ cells loaded with the peptide CD20_p188–196_ or with the peptide with alanine substitution of each amino acid residue (Fig. 3A). Predicted HLA-A*02:01 half maximal inhibitory concentrations (IC_50_s) for alanine-substituted peptides were all below 200 nM, indicating that each alanine-substituted peptide would likely bind to HLA-A*02:01 (table S2). Alanine substitution of the second residue most substantially diminished the affinity of the peptide for HLA-A*02:01 compared to the nonmutated CD20_p188–196_ epitope (table S2), which is consistent with the notion that the second residue of 9-mer peptides often serves as the peptide–HLA-A2 anchor. The non-anchor residues 3, 4, 5, 6, and 7 were inferred to be the residues critical for TCR recognition because alanine substitution of these residues prevented IFNγ production by J1A2 T cells. On the basis of this contact motif information, ScanProsite webtool (*22*) was used to perform in silico search of peptides derived from human proteome that shared the TCR recognition motif “x-x-F-L-G-I-L-x-x,” where x indicates that any one of natural amino acids is accepted in the position. The search resulted in 27 candidate peptides (Fig. 3B and table S3, “Search A”). J1A2 T cells recognized a peptide derived from O56B1 loaded onto HLA-A*02:01^+^ target cells at a supraphysiological concentration (1 μM) (Fig. 3B) (*23*, *24*). However, O56B1 peptide loaded at physiologically relevant concentrations (*23*) did not elicit IFNγ production by J1A2 T cells (Fig. 3C), suggesting that J1A2 T cells are unlikely to cross-react with the O56B1-derived peptide in physiological contexts.

### Amino acid substitution matrix–based peptide library unguided by TCR recognition motif information contained a cross-reactive epitope

**Fig. 4.**
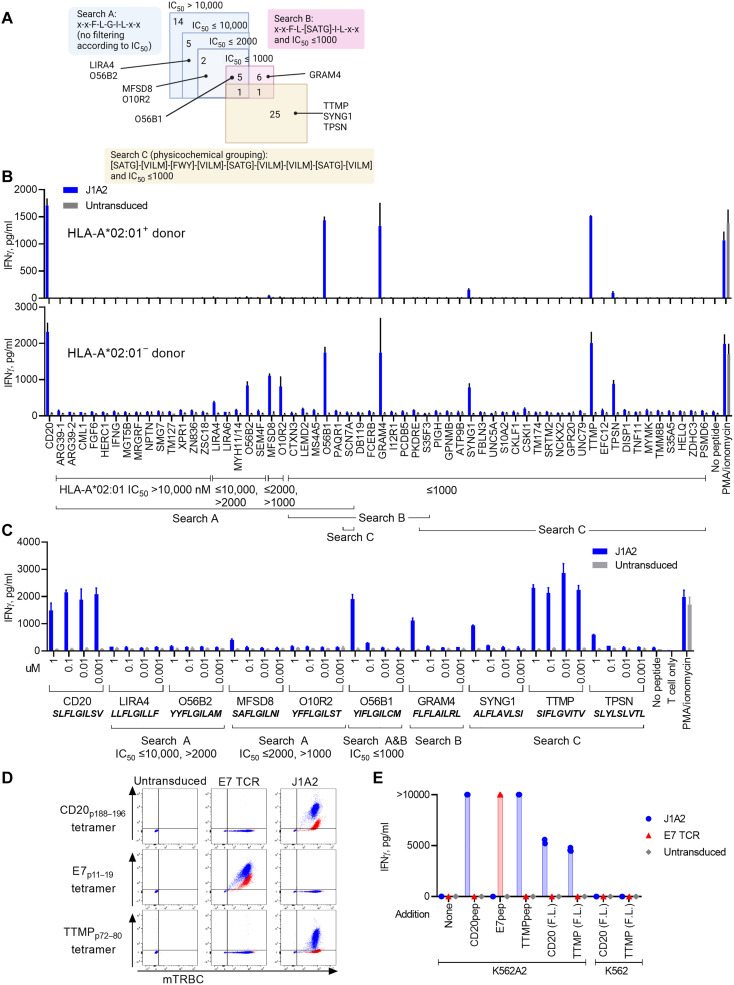
Amino acid substitution–based in silico search identified a cross-reactive epitope. (**A**) Venn diagram shows candidate peptides identified through in silico searches. An in silico search was performed for human peptides that have amino acid sequences that share the TCR recognition motif (Search A), have amino acid sequences that share the TCR recognition motif with conservative substitution of residue 5 with similar amino acids (Search B), or have conservative substitutions of any residues with chemically similar amino acids (Search C). Specific peptides that were subsequently identified to be recognized by the J1A2 TCR at a supraphysiologically high concentration are indicated. (**B**) J1A2 T cells were cocultured with K562A2 loaded with each peptide 1 μM. IFNγ levels of the overnight coculture supernatant were measured with ELISA. The top panel shows a result of J1A2 TCR transduced into HLA-A2^+^ PBMC, and the bottom panel shows a result of J1A2 TCR transduced into HLA-A2^−^ PBMC. (**C**) J1A2 T cells or untransduced T cells were cocultured with K562A2 cells loaded with titrated concentrations of peptides as indicated on the *x* axis. (**D**) E7 TCR T cells, J1A2 T cells, and untransduced T cells were stained with either E7 tetramer, CD20 tetramer, or TTMP tetramer. (**E**) Coculture was set up with K562A2 cells loaded with 1 μM peptides (either CD20_p188–196_, E7_p11–19_, or TTMP_p72–80_) or transduced to express full-length (F.L.) TTMP protein or CD20 protein at an E:T ratio of 1:1 (5 × 10^4^ cells each per well). IFNγ levels of the overnight coculture supernatant were measured with ELISA. Technical replicates: (B) and (C) *n* = 2 and (E) *n* = 3. Representative of (B) four and (C) to (E) two independently performed experiments with independent donor PBMCs.

In silico search criteria were broadened to check for the possibility that there are clinically prohibitive cross-reactive peptides undetected by the established method of cross-reactivity screening against a library of peptides sharing the motif (Fig. 3, B and C). First, given that alanine substitution of residue 5 resulted in more than 90%, but not 100%, reduction in IFNγ production by J1A2 T cells (Fig. 3A), residue 5 may be substituted with similar amino acids. To test this, in silico search was performed for a motif “x-x-F-L-[SATG]-I-L-x-x,” in which residue 5 was allowed to be substituted from glycine to alanine, threonine, and serine based on their similarity of chemical property, hydrophobicity, and size (“Search B” of Fig. 4A and table S3) (*25*, *26*). Furthermore, considering the possibility that cross- reactive peptides may not share the TCR recognition motif sequences, additional in silico search was performed for the following input sequence: [SATG]-[VILM]-[FWY]-[VILM]-[SATG]-[VILM]-[VILM]-[SATG]-[VILM] [“Search C (physicochemical grouping)” of Fig. 4A and table S3]. The amino acids in brackets [] indicate the list of amino acids accepted in the position (*22*). The grouping of amino acids in brackets was based on the physicochemical similarity of amino acids as proposed by The International Immunogenetics Information System (IMGT) and others (*25*–*27*), where [SATG] represent amino acids with very small mass that either are nonpolar or have uncharged side chains, [VILM] represent hydrophobic amino acids with medium-large mass and have either aliphatic or sulfur properties, and [FWY] are aromatic amino acids that are hydrophobic and have very large mass (Table 1, “Physicochemical grouping” column). Proline was omitted from the options of acceptable substitution because of its unique properties to cause kinks (*28*). Last, peptides with predicted HLA-A*02:01 IC_50_ ≥ 1000 nM were filtered out from Search B and C outputs and were not considered for cross-reactivity screening because peptides with low affinity for HLA-A*02:01 are unlikely to result in substantial cross-reactivity, which was supported by the fact that only one peptide with IC_50_ < 1000 nM was recognized by the J1A2 in the initial screening of the TCR against peptides sharing the TCR recognition motif (Search A; Fig. 3B). These additional in silico searches resulted in 6 unique peptides from Search B, 25 unique peptides from Search C, and 1 peptide that was shared by Search B and C results (Fig. 4A).

**Table 1.** Amino acid grouping rules for in silico search.

Amino acid	Physicochemical grouping	BLOSUM62 substitution score ≥ 1	BLOSUM62 substitution score ≥ 0	PAM30 substitution score ≥ 1	PAM30 substitution score ≥ 0
A	[AGST]	[AS]	[ACGSTV]	A	[AS]
C	C	C	[AC]	C	C
D	[DENQ]	[DEN]	[DENQS]	[DEN]	[DEN]
E	[DENQ]	[DEKQ]	[DEHKNQRS]	[DEQ]	[DEQ]
F	[FYW]	[FWY]	[FILMWY]	[FY]	[FY]
G	[AGST]	G	[AGNS]	G	G
H	[RKH]	[HNY]	[EHNRQY]	[HQ]	[HNQ]
I	[VILM]	[ILMV]	[FILMV]	[IV]	[IV]
K	[RKH]	[KEQR]	[EKNQRS]	K	[KR]
L	[VILM]	[ILMV]	[FILMV]	[LM]	[LM]
M	[VILM]	[ILMV]	[FILMQV]	[LM]	[LM]
N	[DENQ]	[DHNS]	[DEGHKNQRST]	[DN]	[DNHS]
P	P	P	P	P	P
Q	[DENQ]	[QKER]	[DEHKMNQRS]	[EHQ]	[EHQ]
R	[RKH]	[KQR]	[EHKNQR]	R	[KR]
S	[AGST]	[ANST]	[ADEGKNQST]	S	[ANST]
T	[AGST]	[TS]	[ANSTV]	T	[TS]
V	[VILM]	[ILMV]	[AILMTV]	[VI]	[VI]
W	[FYW]	[FWY]	[FWY]	W	W
Y	[FYW]	[FHWY]	[FHWY]	[FY]	[FY]

Screening with a high peptide concentration (1 μM) identified as many as nine cross-reactive peptides, although the sensitivity of cross-reactivity detection varied depending on donors (Fig. 4B). Next, the peptides that elicited IFNγ secretion by J1A2 T cells at a high concentration were further evaluated for cross-reactivity with J1A2 T cells at physiologically relevant concentrations. The peptide TTMP_p72–80_ derived from TTMP (also known as C3orf52) from the “Search C (physicochemical grouping) (Fig. 4A)” was recognized by J1A2 T cells at concentrations equivalent to the intended CD20 epitope (Fig. 4C). J1A2 T cells bound to both CD20 tetramers and HLA-A*02:01–TTMP_p72–80_ tetramers (Fig. 4D). Lastly, K562A2 cells transduced with full-length TTMP protein were recognized by J1A2 T cells, confirming that the TTMP-derived peptide is a naturally processed and presented epitope (Fig. 4E). These results collectively demonstrate that J1A2 T cells cross-react with an HLA-A*02:01–restricted epitope of TTMP and highlight that an additional motif-unguided in silico search can uncover cross-reactive epitopes undetected by conventional motif-guided in silico search method.

### Implications of J1A2 TCR cross-reactivity with TTMP-derived peptide

**Fig. 5.**
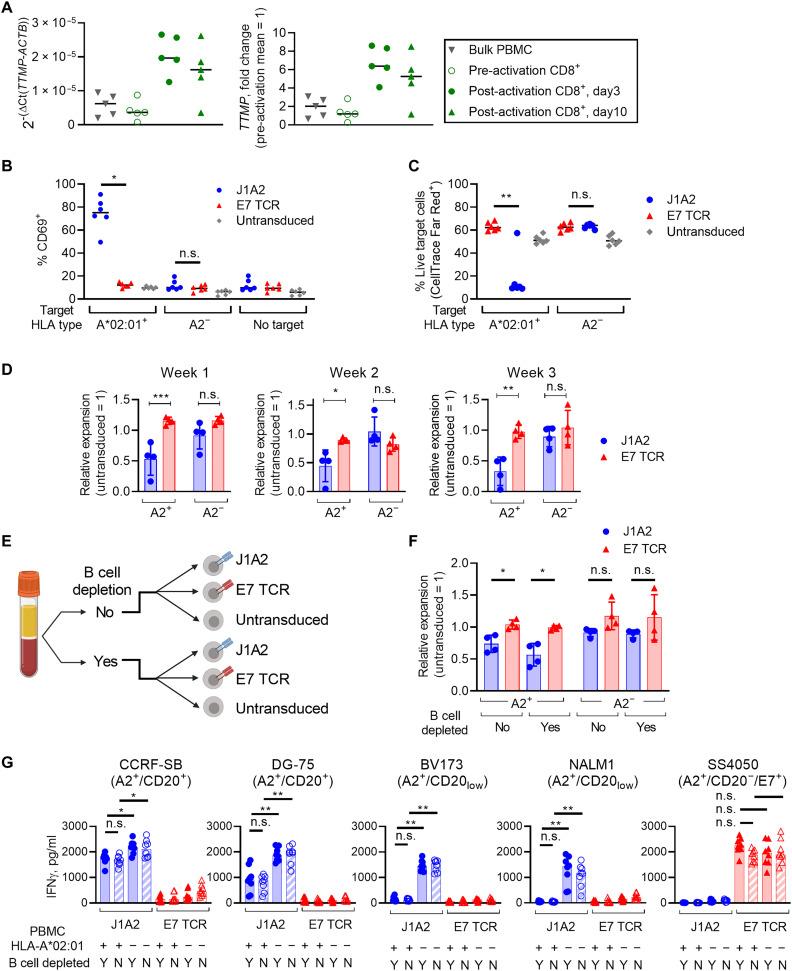
J1A2 T cells recognize HLA-A*02:01^+^ TTMP-expressing activated T cells. (**A**) TTMP gene expression in bulk PBMC, resting CD8^+^ T cells, and CD8^+^ T cells activated with plate-coated anti-CD3 and anti-CD28 antibodies. TTMP expression relative to actin B (left) and fold changes using pre-activation mean as a reference (right) are shown. (**B** and **C**) TCR-transduced T cells (effectors) were cocultured with activated T cells (targets) from HLA-A*02:01^+^ or HLA-A2^−^ donor at an E:T ratio of 1:1, 2 × 10^5^ cells each. Target cells (activated T cells) were labeled with CellTrace Far Red. (B) CD69 expression on effector T cells 1 day after coculture and (C) the fraction of live target cells 2 days after coculture are shown. (**D**) Fold expansion of TCR-transduced T cells at 1, 2, and 3 weeks after TCR transduction. Expansion normalized to untransduced T cells of the same donor is shown. (**E**) Experiment schema. Each donor’s PBMCs were divided, and one-half was depleted of B cells before activation, and the other half was used for experiments without B cell depletion. T cells were transduced with either J1A2 TCR or E7 TCR. (**F**) The expansion of transduced T cells was assessed on week 3 post-transduction. Fold-expansion values normalized to that of untransduced T cells of the same donor are shown. (**G**) TCR-transduced T cells were cocultured with target cell lines at an E:T ratio of 1:1, 5 × 10^4^ cells each. IFNγ levels in the overnight coculture supernatant were measured with ELISA. Statistical significance was determined with Kruskal-Wallis with Dunn’s correction (B), (C), (F), and (G) and ANOVA with Sidak’s correction (D). Biological replicates: (A) *n* = 5, (B) and (C) *n* = 6, (D) and (F) *n* = 4, and (G) *n* = 8. Representative of two independent experiments with independent donor PBMCs. Data are reported as mean ± SD. ***P < 0.001, **P < 0.01, and *P < 0.05; ns, not significant.

TTMP is expressed broadly by various normal tissues [fig. S4A, Human Protein Atlas (proteinatlas.org), and (*29*, *30*)]. While the level of TTMP transcript is low in PBMC and resting CD8^+^ T cells, TTMP expression was augmented by four- to ninefold in T cells after activation (Fig. 5A). T cells are readily accessible tissues and can be easily applied as target cells in experiments that require intact antigen processing and presentation processes. Evaluation of the effect of J1A2 T cells on activated T cells may provide information whether J1A2 T cells could damage vital healthy tissues that express, process, and present the TTMP epitope. First, J1A2 T cells were cocultured with either HLA-A*02:01^+^ or HLA-A2^−^ polyclonal activated T cells as target cells, which were all allogeneic to the J1A2 T cells. J1A2 T cells were activated by and mediated cytotoxicity against HLA-A*02:01^+^ activated T cells (Fig. 5, B and C). Next, the effect of J1A2 T cells on autologous activated T cells was evaluated. Comparison of multiple different donors’ T cells revealed that J1A2 T cells derived from HLA-A*02:01^+^ donors expanded less compared to HLA-A*02:01^−^ donors after transduction of J1A2 TCR (Fig. 5D). It is expected that HLA-A*02:01^+^ B cells would be recognized by J1A2 T cells and may induce terminal differentiation or activation-induced cell death (*31*, *32*) of transduced T cells during the cell manufacturing process, while HLA-A*02:01^−^ B cells would not. To eliminate the possibility that the lower expansion of HLA-A*02:01^+^ J1A2 T cells compared to HLA-A*02:01^−^ counterpart may be explained by the presence of endogenous B cells in the cell products, expansion of TCR-transduced T cells was evaluated with and without depletion of B cells from each donor’s PBMCs before T cell activation and TCR transduction (Fig. 5E). B cells were appropriately undetectable from products that underwent B cell depletion steps (fig. S4B). In HLA-A2^+^ J1A2 T cells, B cells were undetectable even without B cell depletion steps, confirming that J1A2 T cells recognized and eliminated endogenous HLA-A2^+^ B cells (fig. S4B). CD8-to-CD4 ratio of the cell products and transduction efficiency were not significantly different across donors, with or without B cell depletion (fig. S4, C and D). However, the expansion of J1A2 T cells was inferior to E7 TCR T cells or untransduced T cells of the same donor when they were HLA-A2^+^ regardless of whether B cells were depleted before transduction (Fig. 5F). In addition, HLA-A2^+^ J1A2 T cells were functionally less avid compared to HLA-A2^−^ counterpart, as demonstrated by the diminished capacity of HLA-A2^+^ J1A2 T cells to secrete IFNγ upon antigen recognition (Fig. 5G). Because these findings were not mitigated by B cell depletion before TCR transduction, endogenous B cells are unlikely to be the culprit. The data collectively support that both allogeneic and autologous activated T cells that express TTMP but lack CD20 expression are recognized by the J1A2 T cells in an HLA-A*02:01–restricted manner. These observations were unique to J1A2 T cells and were not shared by T cells expressing the TCR against HLA-A*02:01–restricted human papilloma virus type 16 (HPV-16) E7 epitope, E7 TCR T cells, which are clinically active with an established safety profile (*3*). These findings support that J1A2 T cells are cross-reactive against an HLA-A*02:01–restricted epitope of TTMP to a degree in which destruction of vital healthy tissue could occur under normal physiological settings.

### Comparison of different in silico search methods and applicability to other TCRs

In the above example, the detection of the cross-reactive TTMP peptide was achieved using the amino acid substitution rules based on one of conventionally supported classifications (*26*, *27*). There are multiple different and sophisticated matrices that assign similarity scores to each amino acid substitution based on different models (*33*), but there are no empirical data to support superior performance of one amino acid substitution matrix over the other matrices for the purpose of TCR cross-reactive peptide identifications. Alternatively, an extensive approach of CPL scan (*16*–*18*, *34*, *35*) has been used to inform in silico search, which may provide a more comprehensive coverage of potential cross-reactive candidates. However, it is unknown whether the CPL data–guided in silico search is either necessary or optimal for all TCRs in the context of preclinical safety evaluation. Therefore, we next sought to qualitatively compare the performance of different in silico search rules.

**Fig. 6.**
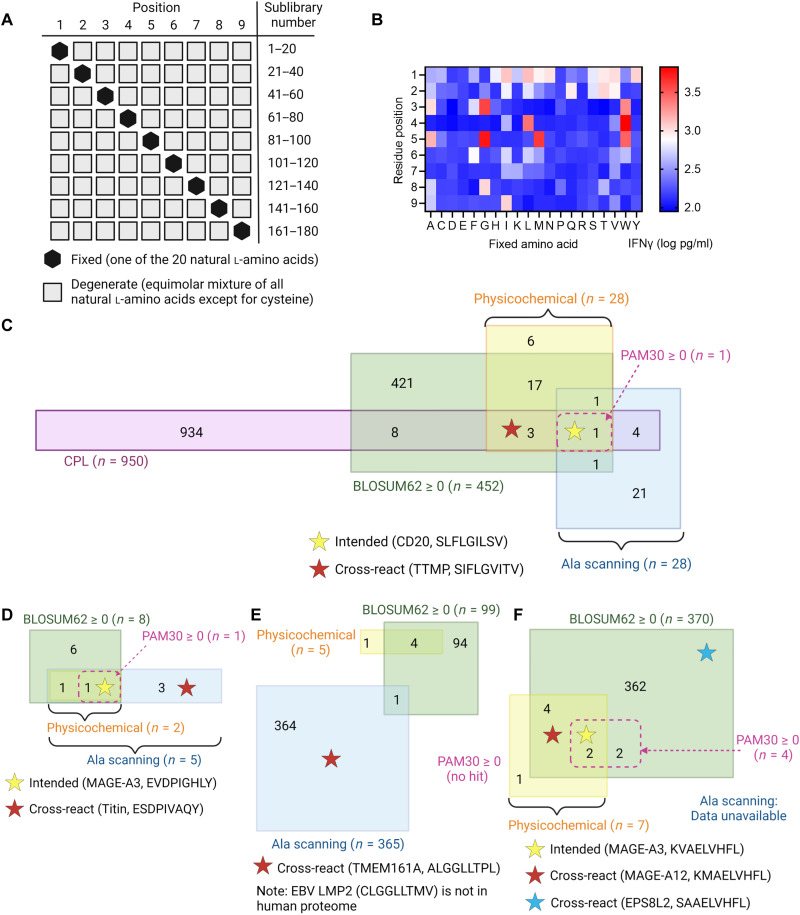
Comparison of different in silico search approaches in CD20 TCR and other TCRs. (**A**) Schema describing the concept of CPL [adapted from (*30*) with permission, Copyright 2010. The American Association of Immunologists Inc.]. (**B**) Heatmap of IFNγ levels from CPL coculture assay. The numerical value on the column is the residue position that was fixed, and alphabet letter on the row is the fixed amino acid in the sublibrary peptides. (**C**) Venn diagram describing the number of unique peptides resulted from each in silico search method. The yellow star denotes the CD20 epitope, the red star symbol denotes the cross-reactive TTMP peptide, and the location of stars indicates which in silico search result did or did not include these peptides. For example, the figure shows that the TTMP peptide was identified with physicochemical grouping, BLOSUM62, and CPL-based in silico search strategies but not with PAM30 or alanine scanning and motif-guided in silico search. Refer to Table 1 for details of each in silico search rules. (**D** to **F**) Venn diagram describing the number of unique peptides resulted from each in silico search method for (D) HLA-A*01:01–restricted MAGE-A3 TCR, (E) HLA-A*02:01–restricted EBV LMP2 TCR, and (F) HLA-A*02:01–restricted MAGE-A3 TCR.

First, to test the performance of CPL data–guided in silico search in our model, CPL scan was executed for the J1A2 T cells. CPL consists of 180 sublibraries, in which each sublibrary contains a mixture of 19^8^ nonamer peptides with a fixed l-amino acid residue in one position, while all other positions have approximately equimolar mixture of all natural l-amino acids (except for cysteine because of its property to form disulfide bonds) [Fig. 6A; adapted from (*30*) with permission, Copyright 2010. The American Association of Immunologists Inc.] (*17*, *35*). There are 20 sublibraries for each fixed residue position because there are 20 natural l-amino acids, amounting to a total of 180 sublibraries for 9-mer CPL (20 sublibraries per fixed residue position × 9 positions = 180 sublibraries). The CPL scan entails coculturing of T cells with target cells loaded with 180 individual sublibraries. J1A2 T cells were cocultured with K562A2 cells loaded with individual sublibrary peptide mixture, and IFNγ levels in overnight coculture supernatant were measured (Fig. 6B and fig. S5). Subsequently, CPL data–guided in silico search was performed using the IFNγ values (mean of four independent biological replicates) of 200 pg/ml from the CPL scan results as the threshold. For the position #8 where the IFNγ level corresponding to the wild-type amino acid was less than 200 pg/ml, any one of natural amino acids was accepted in the position. This definition translated to the search input of [ACFHIKLMNQRSTVWY]-[ACDGILMNQSTV]-[AFGHW]-[ILVW]-[ACGMW]-[FILMVW]-[IKLMV]-x-[AIV] in the ScanProsite tool (*22*). The search resulted in 950 unique peptides, which appropriately included both the CD20 epitope and the TTMP cross-reactive peptide (Fig. 6C and data files S1 and S2). Even after filtering for the peptides with predicted HLA-A*02:01 IC_50_ of 1000 nM or less, there were 420 candidate peptides, which is onerous for manual screening.

Next, motif-guided and motif-unguided in silico search rules using different amino acid substitution matrices, PAM30 and BLOSUM62, were compared (Table 1 and data files S1 to S3). BLOSUM62 matrix–based in silico search resulted in the detection of TTMP peptide with the substitution score threshold of both 0 and 1, but the PAM30 matrix did not yield the TTMP peptide (Fig. 6C and data files S1 and S2). The data show that, in the case of J1A2 TCR, the motif-unguided amino acid substitution–based in silico search provides smaller number of candidate peptides for subsequent screening compared to CPL scan data–guided in silico search. The data also suggest that a certain amino acid substitution matrix may be excessively stringent for the purpose of cross-reactivity screening. The amino acid substitution–based search using either the physicochemical grouping strategy or the BLOSUM62 matrix with threshold substitution score of 1 may be a reasonable starting point to keep the candidate peptide library size relatively small and manageable. Notably, a large candidate peptide list derived from in silico search using the protein BLAST (blast.ncbi.nlm.nih.gov) with lenient parameters did not yield TTMP peptides either (data file S4), highlighting the limitation of the automated protein BLAST approach in the context of cross-reactive peptide identification.

Last, historical TCRs with known cross-reactivity were used to assess whether these in silico search rules apply to other TCRs (Table 2 and data files S1 and S2). Alanine scanning and motif-guided in silico search was necessary to detect the cross-reactive peptide of the TCR intended for HLA-A*01:01–restricted epitope of MAGE-A3 which resulted in off-target cardiac toxicities (Fig. 6D) (*10*, *11*). Similarly, alanine scanning and motif-guided in silico search (fig. S6) was necessary to detect cross-reactivity of an EBV LMP2–specific TCR with a peptide derived from human protein TMEM161A (Fig. 6E) (*36*), although it is unknown whether this cross-reactivity results in toxicities. On the other hand, motif-unguided amino acid substitution–based in silico search was effective at detecting cross-reactivity of the TCR intended for HLA-A*02:01–restricted epitope of MAGE-A3 which resulted in off-target neurological toxicities (Fig. 6F) (*12*). In addition to the culprit cross-reactive epitope of MAGE-A12 (*12*), an additional putative cross-reactive epitope of EPS8L2 was later reported (*37*). Both of these potentially cross-reactive peptides could be identified by motif-unguided amino acid substitution–based in silico search. Interrogation of different TCRs underscores the fact that the method that successfully detects cross-reactivity varies depending on the TCR and that each component has additive value to improve preclinical safety profiles of TCRs being considered for therapeutic purposes.

**Table 2.** Comparison of in silico search rules. Whether in silico search rules resulted in the identification of cross-reactive epitope are indicated as yes, no, or not applicable (N/A) when the data do not exist.

HLA restriction		A*02:01		A*01:01	A*02:01	A*02:01
Intended antigen (epitope amino acid sequence)		MAGE-A3 (KVAELVHFL)		MAGE-A3 (EVDPIGHLY)	CD20 (SLFLGILSV)	EBV LMP2 (CLGGLLTMV)
Cross-reactive antigen (cross-reactive epitope amino acid sequence)		MAGE-A12 (KMAELVHFL)	EPS8L2 (SAAELVHFL)	Titin (ESDPIVAQY)	TTMP (SIFLGVITV)	TMEM161A (ALGGLLTPL)
In silico search rule	Alanine scanning and motif-guided	N/A	N/A	Yes	No	Yes
	Physicochemical grouping	Yes	No	No	Yes	No
	BLOSUM62—0 and above	Yes	Yes	No	Yes	No
	BLOSUM62—1 and above	Yes	No	No	Yes	N/A
	PAM30—0 and above	No	No	No	No	N/A
	PAM30—1 and above	No	No	No	No	N/A
	CPL-scan-data guided	N/A	N/A	N/A	Yes	N/A

## DISCUSSION

**Fig. 7.**
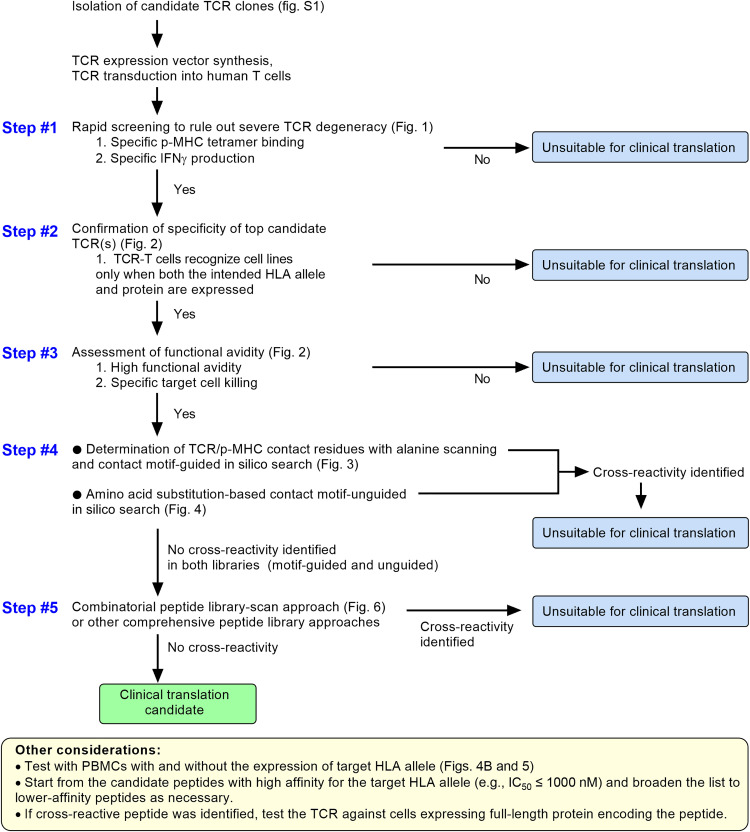
Multi-tiered approach for evaluating TCR cross-reactivity in the context of safety evaluation for TCR-engineered T cell therapy.

This study described systematic screening strategy to prospectively detect clinically prohibitive cross-reactivities of TCRs. A multi-tiered approach of cross-reactivity screening (Fig. 7) as presented in this study can serve as a template for the evaluation of prohibitive cross-reactivity at the preclinical stages of development. This approach is particularly relevant to TCRs that may have supraphysiological affinities, either as a result of affinity enhancement or due to nonautologous sources, such as murine or allogeneic TCR repertoires.

TCRs are structurally biased to interact with MHC and are inherently cross-reactive (*20*, *21*, *34*, *38*, *39*). This makes sense from an evolutionary standpoint given that the theoretical number of possible foreign pathogen sequences exceeds the physiologically possible numbers of unique TCRs and T cells in each individual (*40*). Therefore, the goal of preclinical safety evaluation should be to rule out prohibitive cross-reactivity that would cause clinically relevant tissue damage rather than identifying the TCR with “zero” cross-reactivity, which may not be possible. The J1A2 TCR was cross-reactive against the TTMP_p72–80_ peptide that did not contain the TCR recognition motif of the intended CD20 epitope. The in silico search rule that led to the identification of TTMP_p72–80_ peptide allowed any number of residues at any position to be simultaneously substituted with amino acids considered to be similar. Combining both TCR recognition motif–based search and amino acid similarity–based search that is unguided by recognition motif information, as demonstrated in this report, may represent a sensible strategy to detect cross-reactive peptides. Generally, a TCR would be condemned to be a nonviable therapeutic candidate the moment one clinically consequential cross-reactivity is identified, and whether the TCR also cross-reacts with other peptides is extraneous to the clinical translation decision-making. From this standpoint, the clinical unsuitability of J1A2 TCR was evident even before taking the extensive approach of CPL data–guided in silico search, although screening all candidate peptides from the CPL data–guided in silico search might have identified additional bona fide cross-reactive epitopes of J1A2 TCR. Notably, the overlap of candidate peptides resulted from the CPL data–guided in silico search and other methods were incomplete (Fig. 6C). For example, only three of nine peptides identified to be top candidates from physicochemical grouping method (TTMP, MFSD8, and SYNG1) (Fig. 4C) were detected in the CPL data–guided search output (data file S2). Moreover, estimated concentration of each peptide in a 9-mer CPL peptide pool is 3.1 × 10^−16^ M (*41*), which is likely far lower than the quantitative amount of naturally processed, presented, and successfully targeted cancer-associated antigen (*23*), raising the concern that sensitivity for detecting a particular cross-reactivity may be affected by insufficient concentrations of an individual peptide. This suggests that CPL scan alone may be inadequate for safety evaluation, further emphasizing the importance of multi-tiered approaches (Fig. 7). There are other types of comprehensive peptide library approaches, such as an approach called “X-scan” (*42*–*45*). The peptide library used in X-scan contains target epitope peptide with substitution of each residue one at a time with all possible natural amino acid. In contrast to CPL, X-scan does not take into account the potential effects of simultaneously substituting multiple residues at a time. Comparative analyses of these different peptide library approaches are yet to be performed. Labor-intensive large peptide library–based search may be reserved for the settings where the cross-reactive peptides have not been detected during initial screening against smaller-size peptide libraries. Sophisticated p-MHC display systems (*19*, *46*, *47*) and ever-improving TCR specificity prediction algorithms (*48*, *49*) may also facilitate screening of cross-reactivity in a more high-throughput manner in the future.

Screening for cross-reactivity of TCRs based on target tissue destruction is challenging because there are no tissue panels that adequately cover all HLAs and the human proteome. Therefore, tissue arrays used for the evaluation of cross-reactivity for antibody-based therapies are not applicable to TCR-based therapies. Furthermore, there are no relevant animal models that can address safety of a TCR because no nonhuman animal expresses the human proteome, human antigen processing and presentation machinery, and all possible HLAs. It is also important to note that in vivo HLA immunopeptidome of a given tissue may not always be recapitulated ex vivo. For example, in the study of an affinity-enhanced MAGE-A3 TCR T cells where unexpected off-target cardiac toxicities was reported, a cross-reactive epitope derived from titin was presented by induced pluripotent stem cell (iPSC)–derived beating cardiomyocytes but not by a large set of normal cardiac-derived primary cells (*10*, *11*). However, an iPSC-based approach would be unsuitable for initial high-throughput screening because it requires high sensitivity and broad coverage of the human proteome. Any given cell line expresses only a subset of the human proteome. This may explain why cross-reactivity of J1A2 TCR was not detectable by simply coculturing the TCR-transduced T cells with multiple cell lines (Figs. 1C and 2A and fig. S3). Diminished functional avidity of J1A2 TCR-transduced T cells on the condition of HLA-A*02:01 expression (Fig. 5) was a peculiar observation, which mirrors the report by Duong *et al.* (*50*) who elegantly demonstrated that chronic interactions between TCR and HLA-A2 may induce dysfunctional T cell states and limit antigen-specific responses of T cells expressing high-affinity TCRs against an HLA-A2–restricted epitope of NY-ESO-1. While cross-reactivity was not the focus of their work, it would be interesting to study whether their high-affinity TCRs were also cross-reactive against other HLA-A2–restricted epitopes.

All 10 TCRs against the epitope CD20_p188–196_ discovered in this study were cross-reactive. The TCR affinity threshold required for p-MHC tetramer binding (especially in the absence of CD8 co-receptor engagement) is considered higher than the threshold for T cell activation (*51*). It is possible that the method of isolating T cells from mice based on CD20 tetramer binding might have been biased toward detecting high-affinity TCRs with consequently a high potential for cross-reactivity. Although TCRs against various B cell lineage–restricted antigens, including the same CD20_p188–196_ epitope, have been previously reported in preclinical settings (*52*–*56*), systematic cross-reactivity screening data for those TCRs have not been published. Therefore, it is unknown whether clinically translatable TCRs against the CD20_p188–196_ epitope exist in nature. Murine TCR β chain CDR3 sequences identified in the current study were similar to the published TCR β chain CDR3 motifs of HLA-A*02:01–restricted anti-CD20 TCRs isolated through allogeneic stimulation of human T cells (table S4 and fig. S7) (*55*). The data may imply that T cell responses against this epitope are characterized by the public TCR motif shared across species. It may be reasonable to speculate that CD20_p188–196_ is an epitope against which non–cross-reactive TCRs are difficult to be isolated. The panel of multiple cross-reactive murine TCRs established in the current study could serve as a model to study structural or mechanistic explanations as to why the CD20_p188–196_ epitope has the apparent propensity to elicit cross-reactive TCR responses.

This study has several limitations. First, in silico search was restricted to peptides with nine amino acids in length. Class I restricted epitopes range from 8 to 14 amino acids in length, and it is possible that there are additional cross-reactive peptides of different lengths. Another limitation is that this study focused only on the detection of HLA-A*02:01–restricted cross-reactivity, and it is not designed to detect cross-reactive targets restricted by alternative HLA alleles that are different from the restriction element of the intended target epitope. Although the panel of cell lines used in this work expressed HLA alleles other than HLA-A*02:01 (data file S5), they were far from a comprehensive coverage of all possible HLA alleles in diverse populations. Existence of TCRs that recognize distinct epitopes with different HLA restrictions is evident from the generally accepted fact that some TCRs recognize allogeneic p-MHC complexes. This limitation may be mitigated by adding an approach used in the context of evaluating allogeneic cross-reactivity by testing the T cells against a panel of EBV-LCLs covering HLA alleles commonly shared in populations (*57*). It is unknown whether a similar approach to the current work applies to the cross-reactivity screening of MHC class II–restricted TCRs. There is currently no adequate model to study cross-reactive MHC class II–restricted TCRs because no class II–restricted TCR-transduced T cell therapies have caused clinically significant cross-reactivity to date. It may be theoretically possible, but it is impractical, to screen for combinations of every known HLA allele and peptide sequences in various lengths. Ultimately, only a first-in-human phase 1 clinical trial can determine the clinical safety of a TCR-engineered T cell therapy. Nonetheless, this study provides an example of an effective approach to prospectively detect autoimmune cross-reactivity of therapeutic TCRs and demonstrates complementary nature of each step in the multilayered approach. This strategy is critical to minimize the risks of off-target toxicities of TCR-engineered T cell therapy.

## MATERIALS AND METHODS

### Experimental design

This study was designed to assess different cross-reactivity screening methods using both newly identified TCRs with unknown cross-reactivity potentials and historical TCRs with known cross-reactivities with the goal of providing a practical and efficient framework for evaluating the safety of therapeutic TCRs. TCRs recognizing an HLA-A*02:01–restricted epitope of human CD20 were isolated from HLA-A2 transgenic mice that were vaccinated with the minimal epitope peptide (CD20_p188–196_). TCR α/β paired sequencing was performed using the 10x Genomics platform, and gamma-retroviral expression vector encoding each TCR α/β pair was designed. Human primary T cells transduced with each TCR were first screened for cross-reactivity using p-MHC tetramer binding assays and cell line recognition assays. TCRs that did not demonstrate signs of cross-reactivity during the first screening were subjected to further screenings against candidate human protein–derived peptide libraries. Candidate peptide libraries were generated by conventional contact residue motif–guided search, amino acid substitution matrix–based search unguided by motif information, and CPL scan data–guided search. These methods were compared for their performance at detecting bona fide cross-reactive peptides.

### Mice, tumor cell lines, and human lymphocytes

Mice, cell lines and their sources, and cell culture media are described in Supplementary Methods. All animals were cared in accordance with the protocols approved by the Animal Care and Use Committee at the National Cancer Institute (NCI). Human buffy coats were obtained from healthy volunteer blood donors as by-products of allogeneic blood donation at the National Institutes of Health (NIH) Department of Transfusion Medicine (DTM). Blood donors provided written informed consent for the use of their blood for research purposes, and blood samples were de-identified before distribution from the DTM. De-identified HLA-typed PBMCs in select experiments were obtained through the Center for Immuno-Oncology trial (NCT02821806) at the NCI, in which study participants provided written informed consent. High-resolution HLA class I typing data were available through the NIH Clinical Center HLA laboratory (*9*).

### Vaccination of mice, in vitro T cell stimulation, and screening for CD20-reactive T cells and isolation of p-MHC tetramer^+^ T cells

Vaccination, in vitro T cell stimulation, and isolation of tetramer^+^ cell isolation, including the method of ultraviolet (UV)–mediated ligand exchange and p-MHC tetramer production, are described in Supplementary Methods.

### Single-cell TCR paired sequencing and CDR3 motif analyses

Single-cell TCR α/β paired sequencing using the 10x Genomics platform and all analyses were performed at the NCI Center for Cancer Research Single Cell Analysis Facility, and details are described in Supplementary Methods. The method of CDR3 motif analyses is described in Supplementary Methods.

### Generation of anti-CD20 TCR-transduced T cells

From the single-cell TCR paired sequencing results, TCR clonotypes with V(D)J-spanning productive α/β pairs enriched above the background frequencies were identified. MSGV1 gamma-retroviral vector encoding each TCR was designed. As previously described (*2*), the bicistronic vector was designed to encode TCR β chain, followed by TCR α chain separated by a cleavable linker composed of a furin recognition site and P2A sequences. The mouse TCR constant regions were modified to have an additional interchain disulfide bond and hydrophobic substitutions in the α chain constant region as these modifications have been shown to ensure correct pairing of introduced TCR α/β chains and enhance cell surface TCR expression (*2*, *58*, *59*). Gene synthesis, subcloning, and plasmid preparation were performed by GenScript. Gamma-retroviral supernatant production is described in Supplementary Methods. Human PBMCs were resuspended in T cell media (details in Supplementary Methods). T cells were activated by soluble anti-CD3 antibody (50 ng/ml; clone OKT3, Miltenyi) with recombinant human interleukin-2 (rhIL-2; 300 IU/ml) as previously described (*2*). In Fig. 5 (C to E) and fig. S4 (B to D), PBMCs were first depleted of B cells using human CD19 microbeads (Miltenyi). Following the CD19^+^ cell depletion, T cells were activated with an alternative method to provide costimulatory signals independent of the presence of endogenous antigen-presenting cells: Non–tissue-culture–treated plates were coated with anti-CD3 antibody (1 μg/ml) and anti-CD28 antibody (1 μg/ml; clone CD28.2, BD Biosciences) for 3 hours in 37°C, and then PBMCs were added to the coated plate in T cell media containing rhIL-2 (100 IU/ml). Two days after T cell activation, retronectin-coated non–tissue culture–treated plates were spun with retroviral supernatant for 2 hours at 2000*g* and 32°C. Then, activated T cells were added to the plate. T cells were cultured for a total of 6 to 12 days after the completion of transduction before being used for intended assays.

### Generation of E7 TCR– and EBV LMP2-TCR–transduced T cells

Transduction of E7 TCR and EBV LMP2 TCR was performed using the same procedures as CD20 TCR. E7 TCR was previously described (*2*). EBV LMP2 TCR was isolated from HLA-A*02:01^+^ healthy donor PBMC by repetitive in vitro stimulation of CD8^+^ T cells with the autologous dendritic cells loaded with the target epitope peptide LMP2_p426–434_ (CLGGLLTMV) (Supplementary Methods). EBV LMP2 TCR sequence was determined using the 10x Genomics approach, and the viral vector was reconstructed as described above. The EBV LMP2 TCR used here has the identical CDR3 sequences as one of the published TCRs (*36*).

### Flow cytometry

Antibodies and reagents used for flow cytometry analysis are listed in table S6. Samples were analyzed on BD LSR Fortessa (BD Biosciences) or ACEA NovoCyte Flow Cytometer (ACEA Biosciences, Agilent). FACSDiva and FlowJo version 10 (FlowJo, BD Biosciences) were used for data collection and analysis, respectively.

### Incucyte and morphological documentation of T cell confluency

Details are described in Supplementary Methods.

### Peptides and p-MHC IC_50_ prediction

High-performance liquid chromatography (HPLC)–purified peptides used in this project were synthesized by GenScript and Peptide 2.0 Inc. The HLA-A*02:01 binding predictions were performed on 5 September 2020 using the Immune Epitope Database (IEDB, www.iedb.org) analysis resource tool (*60*) as described in detail in Supplementary Methods.

### T cell functional assays

For the assessment of IFNγ production, T cells were cocultured with target cell lines at cell counts and effector-to-target (E:T) ratio indicated in the figure legend in 96-well round-bottom plate at a final volume of 200 μl per well. After overnight coculture, the supernatant was harvested and the levels of IFNγ were assessed using enzyme-linked immunosorbent assay (ELISA) (kits: IFN-gamma DuoSet ELISA DY285 for human IFNγ and DY485 for mouse IFNγ, R&D Systems; Multiscan FC Microplate Reader, Thermo Fisher Scientific). For in vitro cytotoxicity measurement, target cell lines that are naturally HLA-A*02:01^+^ and CD20^+^ were virally transduced to express firefly luciferase. T cells were cocultured with firefly luciferase–expressing target cell lines at E:T ratios indicated in the figure legend for 6 hours. Upon completion of coculture, luciferin (Promega) was added to the well and the level of luminescence was measured with a luminometer (SpectraMax Microplate Reader, Molecular Devices LLC). For HLA-blocking experiments, target cells were incubated with 50 μg/ml of HLA-blocking antibodies or an isotype control for 3 hours in 37°C, and then T cells were added to each condition as indicated in the figure legend. HLA-blocking antibody clones and sources are listed in table S7.

### In silico search of cross-reactive candidate peptides

ScanProsite webtool was used to perform in silico search of peptides derived from human proteome (*22*). Search input definitions are described in Results, Fig. 4A, and Table 1. Reference protein sequence database selected were UniProtKB/Swiss-Prot including isoforms, and search was restricted only to *Homo sapiens*. ScanProsite was accessed between 1 August 2019 and 2 January 2023, and the latest release number and date of the reference database were “2022_05” and “14-Dec-2022,” respectively. BLOSUM62 matrix (*25*) and PAM30 matrix (*61*) are provided as data file S3. In silico search using protein BLAST is described in Supplementary Methods.

### Quantitative reverse transcription polymerase chain reaction of TTMP

RNA was extracted from PBMC and T cells using the RNeasy Plus Mini Kit (Qiagen), and complementary DNA (cDNA) was synthesized using Invitrogen SuperScript III Reverse Transcriptase (Invitrogen, Thermo Fisher Scientific). For the assessment of other normal human tissues, the cDNA array of normal human tissues was purchased from OriGene. Quantitative polymerase chain reaction (PCR) was performed using the TaqMan Universal PCR Master Mix and the following TaqMan probes: human C3orf52 (TTMP, assay ID Hs01070716_m1) and human ACTB (assay ID Hs99999903_m1). Fold change of TTMP expression before and after T cell activation was calculated using ΔΔCt method.

### CPL scan

CPL was obtained through commercial source (Pepscan) in a lyophilized format. Each sublibrary of CPL was reconstituted with dimethyl sulfoxide to make 20 mM stocks. In a 96-well U-bottom plate, K562A2 cells were plated at 5 × 10^4^ cells per well and incubated with each sublibrary of peptides at 100 μM concentration for 2 hours at 37°C. After peptide loading, J1A2 T cells (5 × 10^4^ cells per well) were added and plates were incubated overnight at 37°C. IFNγ levels in the overnight coculture supernatant were measured with ELISA.

### Statistical analyses

Continuous variables are presented as means ± SD. Mann-Whitney test was used for two-group comparisons. For comparison among groups of three or more, Brown-Forsythe test and Shapiro-Wilk test were used to test the equal variance assumption and normality assumption. When these assumptions held, one-way analysis of variance (ANOVA) was used for one independent variable. Kruskal-Wallis test was used when nonparametric methods were indicated. *P* values were adjusted for multiple comparisons, using Holm-Sidak method (for one-way ANOVA) or Dunn’s methods (for Kruskal-Wallis). Two-tailed *P* values of <0.05 were considered significant. GraphPad Prism version 9 (GraphPad Software) was used for analyses.

## Supplementary Materials

### This PDF file includes:

Supplementary Methods Figs. S1 to S7 Tables S1 to S7 References
