# Supplementary material for: Multi-tiered approach to detect autoimmune cross-reactivity of therapeutic T cell receptors
Source: Sci Adv. 2023 Jul 26;9(30):eadg9845. doi: 10.1126/sciadv.adg9845 (PMC10371023; doi:10.1126/sciadv.adg9845)
Supplement: Supplementary file 1 — Supplementary Methods Figs. S1 to S7 Tables S1 to S7 References [file sciadv.adg9845_sm.pdf]

Science Advances
AAAS

Supplementary Materials for

Multi-tiered approach to detect autoimmune cross-reactivity of therapeutic T cell receptors

Kazusa Ishii *et al.*

Corresponding author: Kazusa Ishii, kazusa.ishii@nih.gov; Christian S. Hinrichs, ch977@cinj.rutgers.edu

Sci. Adv. **9**, eadg9845 (2023)
DOI: 10.1126/sciadv.adg9845

The PDF file includes:

Supplementary Methods
Figs. S1 to S7
Tables S1 to S7
References

Other Supplementary Material for this manuscript includes the following:

Data files S1 to S5

Supplementary Methods

Mice

B6.HLA-A2 (C57BL/6-Mcph1$^{Tg(HLA-A2.1)1Enge}$/J) and B6.AireKO (B6.129S2-Aire$^{tm1.1Doi}$/J) were purchased from Jackson Laboratory. B6-HLA-A2 mice were crossed with B6.AireKO to produce B6-HLA-A2 with homozygous deletion of autoimmune regulator (*Aire*) (B6.HLA-A2-AireKO). Genotyping of *Aire* gene was performed using standard PCR technique. Primer sequences are provided in the Supplementary Table S5. All animals were cared in accordance with the protocols approved by the Animal Care and Use Committee at the National Cancer Institute.

Cell lines

K562, RPMI1788, RPMI6666, CCRF-SB, JVM-2, NALM-1, DG-75, SU-DHL-4, DB, JeKo-1, Raji, Ramos, NALM6, THP-1, CaSki, and MOLT-4 were purchased from ATCC. Cell line BV-173 was purchased from DSMZ. Cell lines were tested negative for mycoplasma using the Mycoplasma PCR Detection Kit (Applied Biological Materials Inc.) Production of EBV-LCL and HLA typing were previously described (9).

Cell culture media and incubator condition

Human leukemia, lymphoma, and cancer cell lines used in this projects were all cultured in RPMI 1640 with 10% heat-inactivated fetal calf serum (Omega Scientific), L-alanyl-L-glutamine dipeptide (Gibco, Life Technologies, GlutaMAX diluted to 1x), nonessential amino acids (Gibco, Life Technologies, MEM-NEAA diluted to 1x), sodium pyruvate 1 mM (Gibco, Life Technologies), HEPES 15 mM (Gibco, Life Technologies), penicillin 100 units/mL and streptomycin 100 μg/mL (Gibco, Life Technologies). For culturing of murine T cells, the same RPMI 1640-based media was further supplemented with 2-mercaptoethanol 50 μM (Gibco, Life Technologies) and recombinant human IL-2 (rh-IL-2) 30 IU/mL (Aldesleukin, Prometheus). For human T cells, we used T cell media, which consisted of AIM-V media (Gibco, Life Technologies) supplemented with 5% heat-inactivated fetal calf serum, L-alanyl-L-glutamine dipeptide (Gibco, Life Technologies, GlutaMAX diluted to 1x), nonessential amino acids (Gibco, Life Technologies, MEM-NEAA diluted to 1x), sodium pyruvate 1 mM (Gibco, Life Technologies), HEPES 15 mM (Gibco, Life Technologies), penicillin 100 units/mL and streptomycin 100 μg/mL (Gibco, Life Technologies), and gentamycin 50 ug/mL (Gibo, Life Technologies). All cultures were performed in humidified incubator at 37°C with 5% CO2.

UV-activated peptide exchange and p-MHC tetramer synthesis

UV-mediated ligand exchange and p-MHC tetramer production were performed as previously described (62) using Flex-T HLA-A*02:01 Monomer UVX (BioLegend), streptavidin-PE (Life Technologies), and a UV lamp (Camag).

*Mouse vaccination and identification of T cell populations recognizing HLA-A*02:01-restricted epitope of human CD20*

The 9 mer peptide CD20$_{p188\text{-}196}$ (SLFLGILSV) of human CD20 protein has been reported to be an HLA-A*02:01-restricted epitope (52, 53, 55, 56). To establish a panel of multiple murine TCRs with a specificity toward human CD20, a human self-antigen, B6.HLA-A2 mice were vaccinated with the

peptide $CD20_{p188-196}$ along with poly(I:C) and anti-mouse CD40 monoclonal antibody (Supplementary Figure S1A). Female mice between 6 and 10 weeks of age were intraperitoneally injected with a mixture of the $CD20_{p188-196}$ peptide 100 µg (>95% purity, Peptide 2.0, Inc and Genscript), poly(I:C) 50 µg (InvivoGen), and anti-CD40 monoclonal antibody (clone FGK4.5, BioXcell) in PBS adjusted to a final total volume of 200 µL per mouse on days 0, 14, and day 28 (anti-CD40 monoclonal antibody was omitted from day 28). Peripheral blood was collected on days 7, 21, and 35 to assess for circulating tetramer-binding T cell frequencies. Chronological changes in T cell responses were assessed by obtaining peripheral blood from each mouse between scheduled vaccinations. Additionally, CD20 tetramers+ CD8+ T cells were isolated from B6.HLA-A2 mice with homozygous deletion of autoimmune regulator (*Aire*) (B6.HLA-A2-AireKO) using the same vaccination method. The *Aire* gene encodes a protein that regulates tissue-specific antigen expression in medullary thymic epithelial cells (63), playing a critical role in central tolerance. Therefore, *Aire* deficient mice are assumed to have a different underlying TCR repertoire compared to *Aire* competent mice, although murine CD20 or the mouse proteome do not contain the intended epitope sequence from human CD20.

Murine T-cell in vitro T-cell stimulation

Following vaccinations, mice were humanely euthanized and lymphoid tissues were harvested on day 35. Red blood cells were lysed using ACK Lysing Buffer (Lonza) and single cell suspension was filtered through sterile 40 µm filter. Cells were used for *in vitro* T-cell simulation: Cells were re-suspended in RPMI 1640-based media (as described above) with an addition of 2-mercaptoethanol 50 µM, recombinant human IL-2 (rh-IL-2) 30 IU/mL, and the $CD20_{p188-196}$ peptide 1 µM. Cells were plated in tissue-culture treated 24-well plate, 6e6 cells/well in 2 mL volume, and cultured for 1 week in 37°C. On day 42 (i.e. a week after *in vitro* stimulation) cells were re-analyzed on flow cytometry or used in co-culture assays. Mouse splenocytes were incubated with HLA-A*02:01-$CD20_{p188-196}$ tetramer conjugated with PE (1 µL of p-MHC tetramer per 1e6 T cells in 10 µL MACS buffer) on ice for 30 minutes protected from light. Then, Anti-PE-MicroBeads (Miltenyi) was used to perform positive selection of PE+ population following the manufacture's instruction.

T cells bound to CD20 tetramers were magnetically isolated from mouse splenocytes (either immediately following tissue harvest or after *in vitro* stimulation), and the paired TCR α chain and β chain nucleotide sequences at a single cell level were determined using the 10x Genomics platform.

Isolation of EBV LMP2-specific TCR from human PBMC

From HLA-A*02:01+ human PBMC, monocyte-derived dendritic cells were generated using adherence technique. Briefly, PBMC were resuspended in CellGenix GMP DC medum (Cellgenix) with 1% heat-inactivated human AB serum (HS) (Gemini Bio) in 6-well TC-treated plate at 1 e7 cells/well. Following 90 minutes of incubation in 37°C, non-adherent cells were gently removed by swirling and pipetting with warm serum-free media. Cells adherent on the bottom of the plate representing monocytes were cultured for overnight with Cellgenix DC media, 1% HS with IL-4 1000 IU/mL and GM-CSF 800 IU/mL to make immature dendritic cells (DCs). The next morning, following cytokines were added to the culture to achieve final concentrations of TNFα 10 ng/mL, IL-1β 10 ng/mL, IL-6 1000 IU/mL, and PGE2 1 µg/mL. Approximately 24 hours later, matured DC were harvested and loaded with minimal epitope peptide (CLGGLLTMV) at 1 µM for 30-60 minutes in 37°C, irradiated with 40 Gy, and co-cultured with autologous CD8+ T cells (autologous to the DC) at an E:T = 10:1, CD8+ 1e6 cells and DC 1e5 cells/well in 48-well TC-treated plate, in RPMI-based media (RPMI, 10% HS, L-alanyl-L-glutamine dipeptide and

penicillin / streptomycin 100 μg/mL) in the presence of IL-21 30 ng/mL. Three days later, IL-7 10 ng/mL, IL-2 25 IU/mL, and IL-15 2 ng/mL were added in the co-culture media. Following 7-14 days of co-culture, the presence of EBV LMP2-tetramer binding CD8+ T cells were assessed with flow cytometry analysis, and tetramer binding cells were isolated and used for TCR sequencing. *In vitro* stimulation of CD8+ T cells with autologous PBMC were repeated as necessary.

Single cell TCR paired sequencing

Single cell TCR alpha/beta paired sequencing and all analyses were performed at the National Cancer Institute Center for Cancer Research Single Cell Analysis Facility. This work utilized the computational resources of the NIH HPC Biowulf cluster. (http://hpc.nih.gov). From the samples that yielded productive V(D)J spanning pairs with clonotype enrichment, 10 unique TCRs were identified (Supplementary Table S1).

Single Cell – Partitioning and Library Preparation: Single cell suspensions of T cells stored in RNA Protect (Qiagen) were aliquoted 25ul each into several microcentrifuge tubes and volumed up to 1mL with PBS. These were centrifuged at 300g for 5mins, supernatant removed, and then cells were resuspended in approximately 50uL of remaining buffer volume. Cell counts were measured using a fluorescent cell counter with propidium iodide and acridine orange (LunaFL, Logos Biosystems). Cell suspensions were loaded at maximum volume onto the 10x Genomics Chromium platform using the 5' v1.1 immune profiling chemistry (10x Genomics). Following partitions, reverse transcription, and cDNA amplification, mouse TCR enrichment libraries were prepared according to vendor recommendations.

Single Cell – Sequencing: Sequencing of single cell mouse TCR libraries were performed on an Illumina NextSeq 550/500 instrument with paired end 150bp reads and an 8bp read for sample indices. Samples were multiplexed for sequencing and reads were combined from multiple sequencing runs to achieve at least 10,000 reads per cell on average for TCR libraries for all samples.

Single Cell – Data Processing: Data was processed using the 10x Genomics cellranger pipeline (v3.1.0) to demultiplex reads and then align reads to a GRCm38 VDJ reference for single cell TCR data (refdata-cellranger-vdj-GRCm38-alts-ensembl-3.1.0). UMI-adjusted aligned reads were used to generate a TCR clonotype matrix that was used for downstream analysis.

CDR3 motif analysis

An R package "ggseqlogo" was used to generate position weight matrix of CDR3 amino acid sequences (64). The '-' character was used as a place holder to align CDR3 amino acid sequences of different lengths across multiple TCR clonotypes.

Gamma-retroviral supernatant production

Gamma-retroviral supernatant production was performed by co-transfection of 293GP cells with a plasmid encoding the envelope RD114 and the retroviral (MSGV) TCR expression vectors using the lipofectamine 3000 (Life Technologies) following the manufacturer's protocol. Viral supernatant was collected 48, 72 and 96 hours after the transfection and frozen and stored in -80°C until use.

Peptides and Peptide-MHC IC50 prediction

HPLC-purified peptides used in this project were synthesized by Genscript and Peptide 2.0 Inc. The HLA-A*02:01 binding predictions were performed on 9/5/2020 using the IEDB (www.iedb.org) analysis resource Consensus tool (60). As previously described in the IEDB website, the Consensus tool combines predictions from ANN aka NetMHC (4.0) (65-67), SMM (68) and Comblib (69).

Incucyte

Confluency of transduced T cells were monitored with Incucyte S3 (Essen BioScience, Sartorius). Five days after the transduction and the confirmation of transduction efficiency, TCR-transduced T cells were counted and plated at 2.5 e5 cells/well in 2 mL volume in TC-treated 24-well plate. Phase contrast images were taken, and confluency was calculated using the Incucyte software (Incucyte 2022B Rev1) following the manufacture's protocol.

In silico search using the BLAST

Protein BLAST was accessed on April 12, 2021, May 1, 2021, November 28, 2022, and December 14, 2022 (70, 71). Query sequence was the $CD20_{p188\text{-}196}$ peptide sequence (SIFLGILSV) and the search database was set as "Non-redundant protein sequences (nr)" with a filter for homo sapiens (taxid:9606). We chose the "blastp" algorithm and set the algorithm parameters as follows: max target sequences of 1000, check the box for "automatically adjust parameters for short input sequences" which led the blastp program to automatically use PAM30 matrix, gapcosts of 9/1, word size 2, and expect value of 200,000.

References cited in the supplementary materials are in the main reference list in the main text.

Supplementary Figure S1

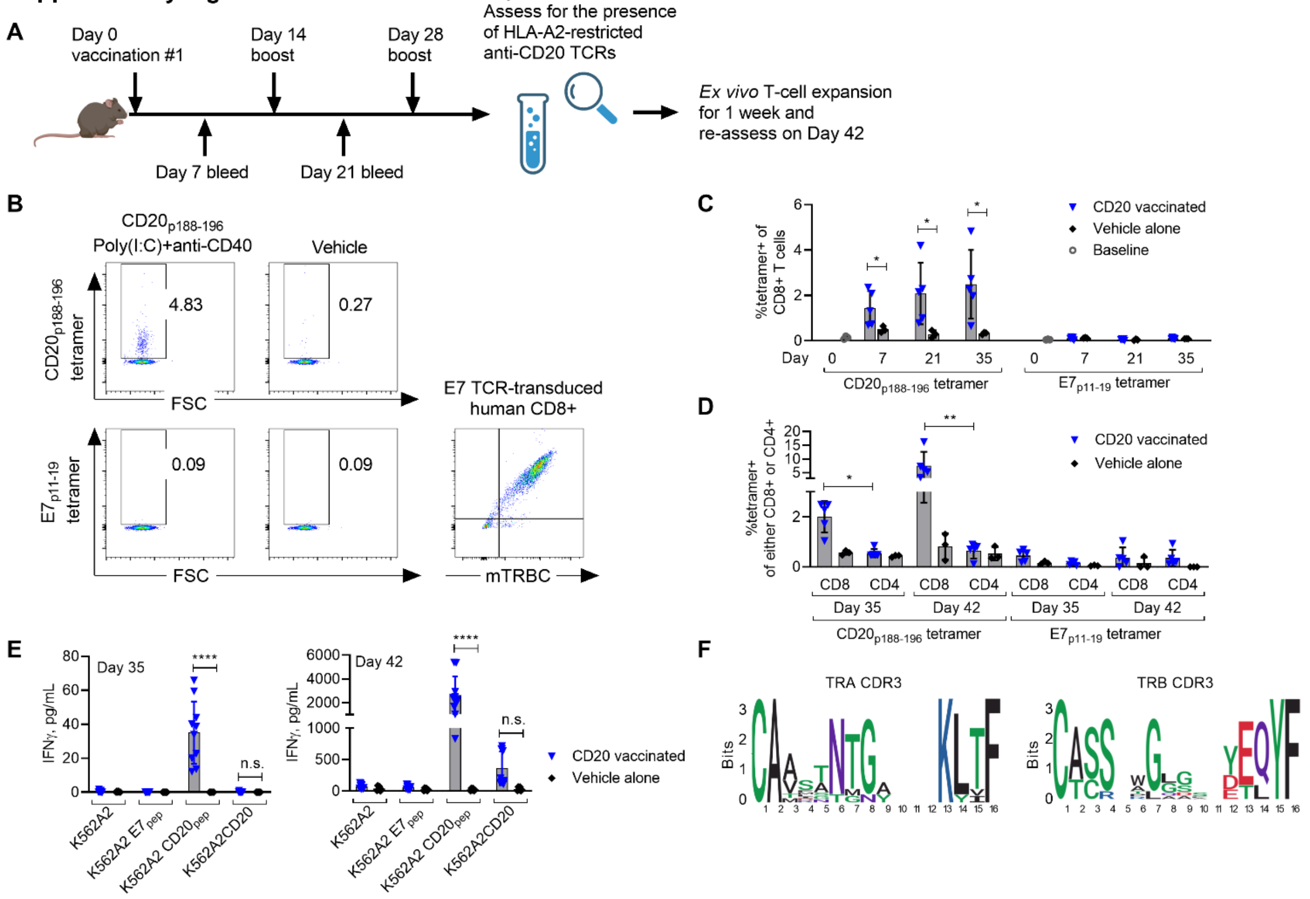

Supplementary Figure S1. Isolation of murine TCRs recognizing the HLA-A2-restricted epitope of human CD20:

(**A**) Vaccination schema. B6.HLA-A2 mice were intraperitoneally injected with minimal epitope peptide $CD20_{p188-196}$ 100 ug, poly(I:C) 50 ug, and anti-CD40 monoclonal antibody 50 ug on days 0, 14, and day 28 (anti-CD40 monoclonal antibody was omitted from day 28). Peripheral blood was collected on days 7 and 21 to assess for peptide-specific T-cell responses. A week after the last boost vaccination, lymphoid tissues were harvested. A portion of mouse splenocytes and circulating lymphocytes were stimulated *in vitro* by culturing in the presence of $CD20_{p188-196}$ peptide 1 uM. A week after *in vitro* stimulation (day 42), tetramer-binding was reassessed. (**B**) Representative FACS dot plots gated on CD8+ T cells in PBMC collected on day 35. PBMC were stained with respective HLA-A2-pepide tetramers. E7 TCR transduced T cells were used as E7 tetramer staining positive controls. Murine TCR beta constant region (mTRBC) was used as a marker of E7 TCR transduction efficiency. (**C**) The percentage of tetramer-binding population within CD8+ T cells in PBMC. (**D**) The percentages of tetramer-binding population in splenocytes were assessed with flow cytometry on day 35 (immediately following spleen harvest) and day 42 (one week after *in vitro* stimulation). (**E**) Splenocytes were co-cultured with target cell lines. IFNγ levels of the overnight co-culture supernatant were measured using ELISA. E:T ratio on day 35 was 20:1 (polyclonal splenocytes 1e6 cells with target cell lines 5e4 cells) and on day 42 was 5:1 (2.5e5 splenocytes with target cell lines 5e4 cells) in a 96-well round-bottom plate for a final volume of 200 uL/well. K562A2 is K562 retrovirally transduced to express HLA-A*02:01; K562A2 E7pep and K562A2 CD20pep indicate K562A2 loaded with 1uM of peptide $E7_{p11-19}$ and $CD20_{p188-196}$, respectively; K562A2CD20 is K562A2

transduced with full-length CD20 protein. (**F**) Position weight matrix of TCR alpha (TRA) and TCR beta (TRB) chain CDR3 sequences. Statistical significance was determined using Mann-Whitney test (C) and Kruskal-Wallis with Dunn's correction (D, E). Data are reported as mean +/- SD. n = 3-5 (C, D); n = 10 (E). ****$P<0.0001$, **$P<0.01$, *$P<0.05$, ns = not significant.

Supplementary Figure S2

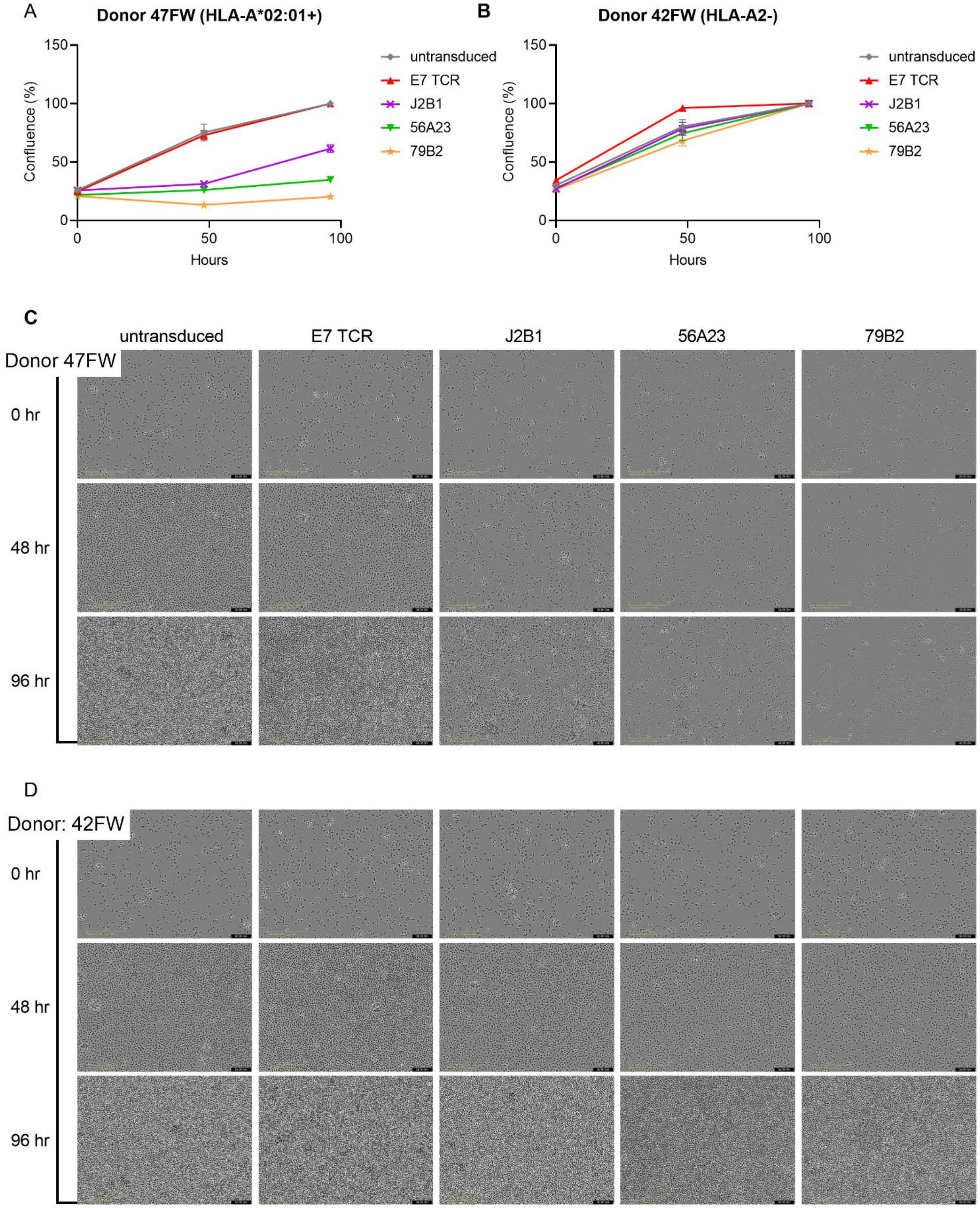

Supplementary Figure S2. Fratricide of TCR-transdcued T cells:

Transduced T cells were plated in 24-well TC-treated plate at the initial seading desity of 2.5e5 cells/well. Confluency of cells were motitored for 4 days using the Incucyte S3. (**A, B**) Confluency calculated using the Incucyte analysis software and (**C, D**) phase contrast images are shown. Scale bar in each phase contrast image is 200 μm.

Supplementary Figure S3

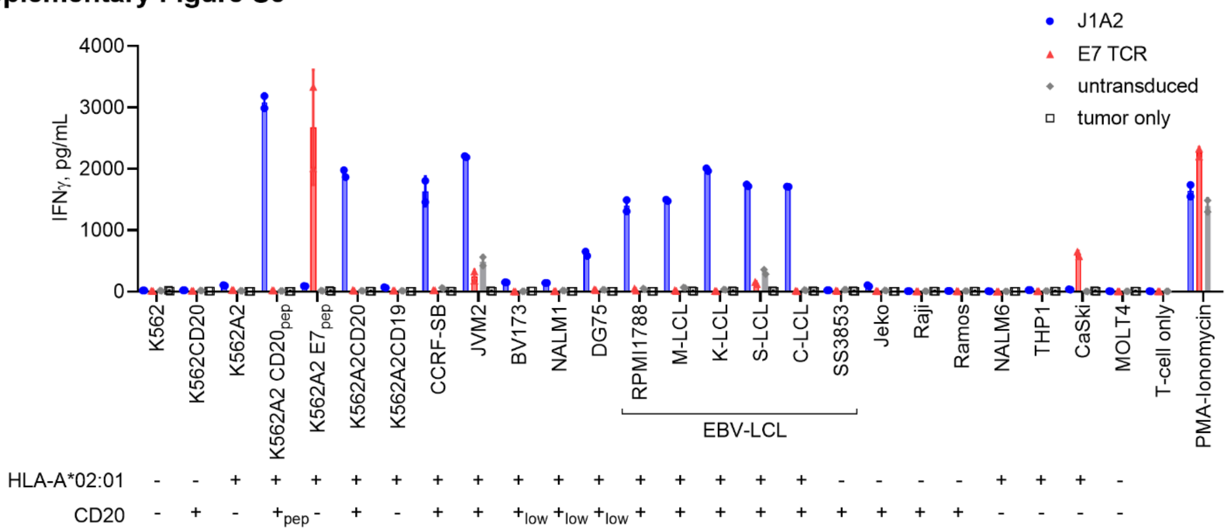

Supplementary Figure S3. J1A2 TCR T cells recognize target cell lines naturally expressing both HLA-A*02:01 and CD20:

J1A2 TCR-transduced T cells, E7 TCR-transduced T cells, or untransduced T cells were co-cultured with target cell lines indicated in the x-axis at an E:T ratio of 1:1 (5e4 cells each) in 96-well U-bottom plate overnight. Co-culture supernatant was harvested and IFNγ levels were assessed with ELISA. Data shown is from a representative donor with two technical replicates.

Supplementary Figure S4

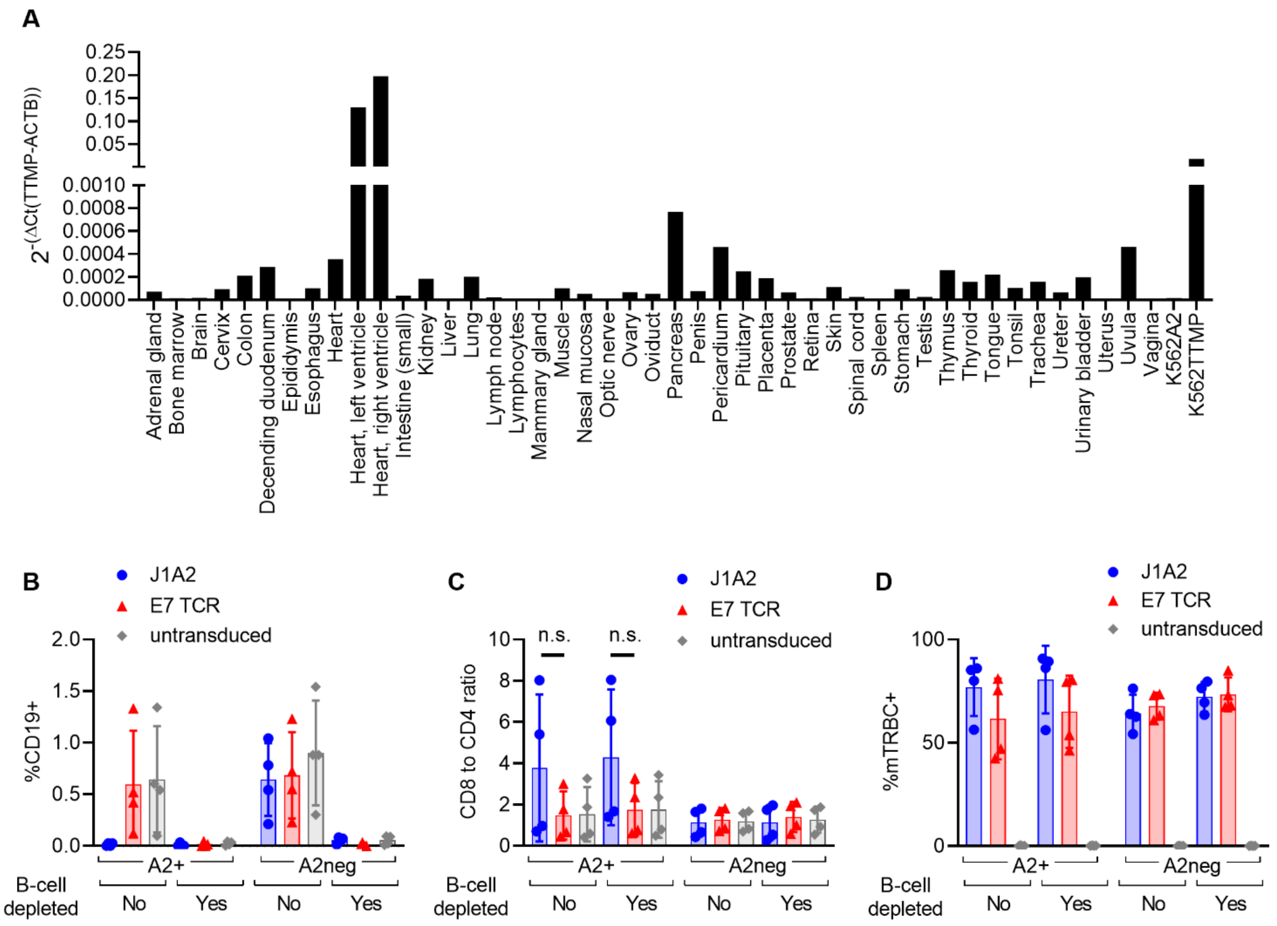

Supplementary Figure S4. TTMP gene expression by normal tissues and comparison of HLA-A*02:01+ J1A2 T cells to HLA-A2- counterpart:

(**A**) Normal human tissue cDNA array was used to assess TTMP gene expression. TTMP transcripts relative to actin B are shown. K562TTMP is K562 cell line transduced with TTMP, uses as a positive control. The figure is a representative of two independent assays using two independent cDNA array plates. There were no technical replicates. (**B-D**) As described in the main Figure 5C, T cells were transduced with TCR with or without B-cell depletion from the starting PBMC material. (**B**) CD19+ cells in cell products assess on 5 days after the completion of transduction. (**C**) CD8 and CD4 phenotype of cell products were assessed on day 5 after the completion of transduction. Y-axis is a ratio of CD8 over CD4. (**D**) Transduction efficiency was assessed using murine TCR beta constant region (mTRBC) as a marker. n = 4 (B-D). Kruskal-Wallis with Dunn's correction (C).

Supplementary Figure S5

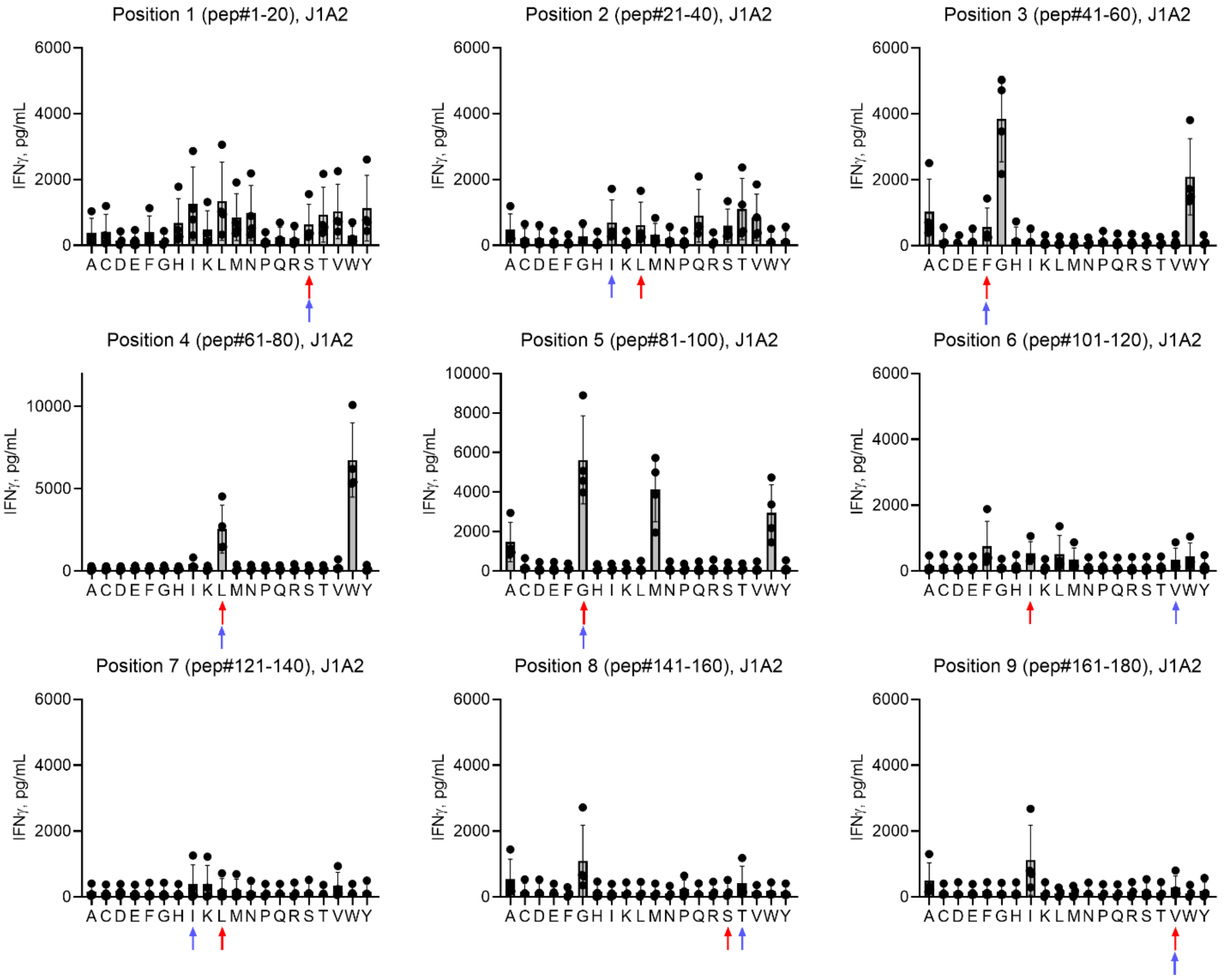

Supplementary Figure S5. Combinatorial peptide library scanning:

J1A2 T cells were co-cultured with K562A2 cells loaded with each sublibrary of combinatorial peptide library100 μM. IFNγ levels in overnight co-culture supernatant was measured. Pooled results from 4 independent biological replicates are shown. Red arrow indicates the amino acid of $CD20_{p188-196}$ (SLFLGILSV) in the position. For example, in the “Position 1” graph, red arrow highlights S because the first residue of the CD20 epitope is serine. Similarly, blue arrow indicates the amino acid of $TTMP_{p72-80}$ (SIFLGVITV) in the position.

Supplementary Figure S6

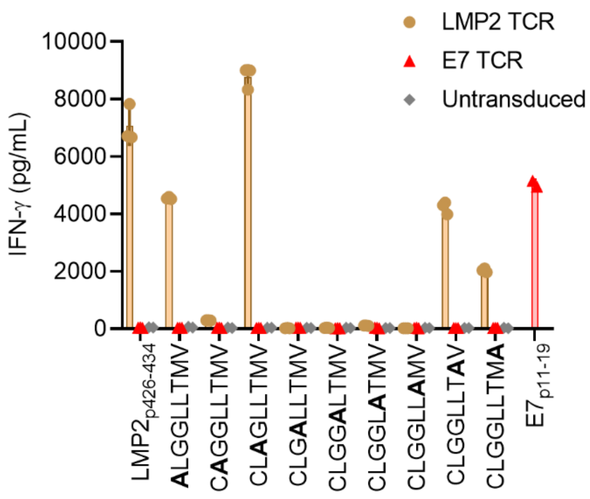

Supplementary Figure S6. Alanine scanning of EBV LMP2 TCR:

EBV LMP2 TCR T cells were co-cultured with K562A2 cells loaded either with the wild-type EBV $LMP2_{p426-434}$ peptide or with peptides with alanine substitution on each residue at 1 μM. IFNγ levels in the overnight co-culture supernatant was measured with ELISA. Second and nineth residues are HLA-A*02:01 anchor residues. Based on the alanine scanning result, residues on positions 4, 5, 6, and 7 were inferred to be the non-anchor residues important for TCR recognition. Contact motif-guided in silico search rule in ScanProsite was x-x-x-G-L-L-T-x-x.

Supplementary Figure S7

A Position weighted matrix of murine TCR β CDR3s isolated in this project

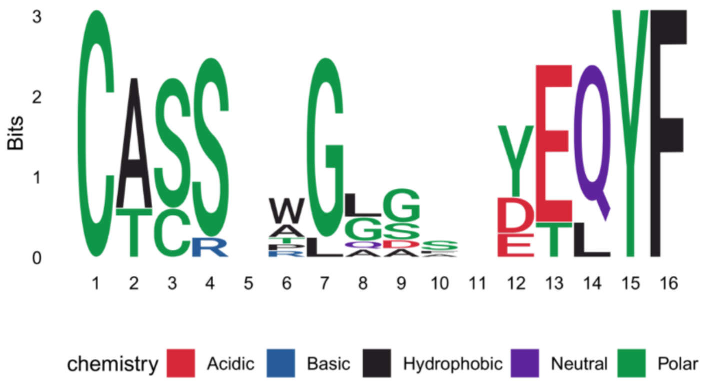

B. Position weighted matrix of human TCR β CDR3s reported by Abrahamsen IW et al. (Leukemia 2010)

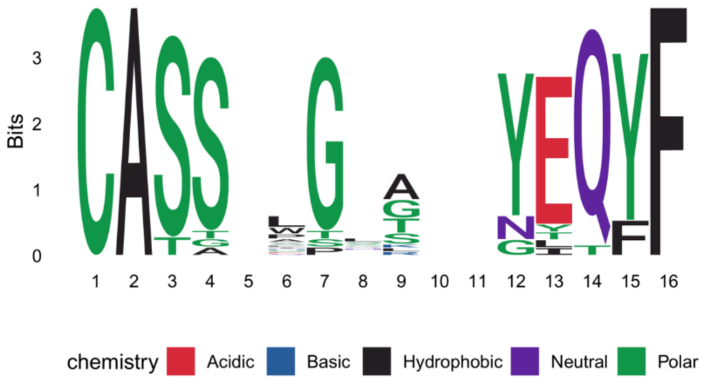

C. Position weighted matrix generated by combining both the murine TCR β CDR3 sequences from this project and published human TCR β CDR3 sequences

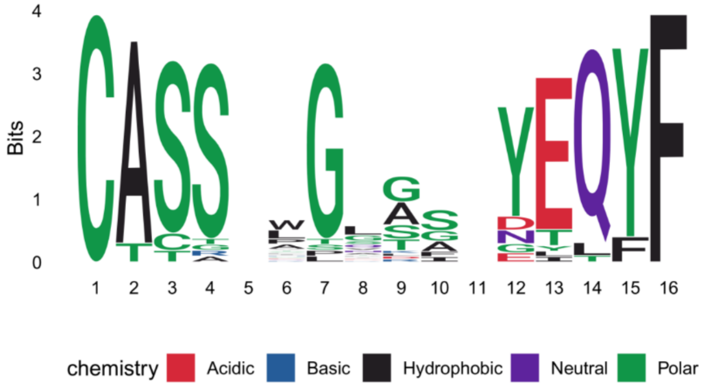

Supplementary Figure S7. TCRs recognizing HLA-A*02:01-restricted epitope of CD20 derived from mice and humans share TCRβ motif:

Position weighted matrices of TCRβ CDR3 sequences were generated for (A) murine TCRβ CDR3 sequences isolated in this project (identical to the main Figure 1E), (B) human TCR CDR3 sequences reported by Abrahamsen IW et al. (*Leukemia* 2010), and (C) combining murine TCRβ CDR3 sequences (A) and previously reported human TCR CDR3 sequences (B).

Supplementary Table S1: TCR clonotypes identified from vaccinated mice

Mouse strain	Clone	TRBV	TRBJ	TRB, CDR3	TRAV	TRAJ	TRA, CDR3
B6.HLA-A2	J1A1	1*01	2-7*01	CTCSTGDEQYF	7D-2*02	27*01	CAASTNTGKLTF
B6.HLA-A2	J1A2	17*01	2-7*01	CASSPGASYEQYF	7-6*02	40*01	CAVFNTGNYKYVF
B6.HLA-A2	J1A3*	1*01	2-7*01	CTCSAGDEQYF	7D-2*02	27*01	CAASTNTGKLTF
B6.HLA-A2.*Aire-*	869A1	19*01	2-7*01	CASGGAFSYEQYF	7-5*01	27*01	CAVITNTGKLTF
B6.HLA-A2.*Aire-*	869A2*	1*01	2-7*01	CTCSAGDEQYF	7D-2*02	27*01	CAASTNTGKLTF
B6.HLA-A2	J2B1	4*01	2-7*01	CASSWGLGYEQYF	14-2*02	52*01	CAASANTGANTGKLTF
B6.HLA-A2.*Aire-*	56A22	13-3*01	2-3*01	CASSDRLGGSAETLYF	3-1*01	27*01	CAVDTNTGKLTF
B6.HLA-A2.*Aire-*	56A23	29*01	2-7*01	CASSWGQGYEQYF	4D-3*01	52*01	CAALANTGANTGKLTF
B6.HLA-A2.*Aire-*	56B2	14*01	2-7*01	CASSLSSYEQYF	13N-1*01	9*01	CAMMSNMGYKLTF
B6.HLA-A2.*Aire-*	56B8	4*01	2-3*01	CASRPWGGDAETLYF	8D-2*02	23*01	CATRGKLIF
B6.HLA-A2.*Aire-*	79B2	4*01	2-7*01	CASSWGLGSYEQYF	5D-4*02	52*01	CAASANTGANTGKLTF

*Clonotypes J1A3 and 869A2 had identical TCR α chain and β chain sequences.

Supplementary Table S2: Predicted HLA-A*02:01 IC50 of $CD20_{p188\text{-}196}$ epitope and alanine-substituted peptides.

Peptide amino acid sequences	IC50 (nM)
SLFLGILSV	7.7
ALFLGILSV	8.6
SAFLGILSV	142.7
SLALGILSV	14.2
SLFAGILSV	3.3
SLFLAILSV	13.8
SLFLGALSV	11.8
SLFLGIASV	8.1
SLFLGILAV	8.3
SLFLGILSA	26.1

Supplementary Table S3: List of candidate peptides for cross-reactivity screening

Protein	amino acid sequence	Search method
ARG39, p46-54	TYFLGILKA	A
ARG39, p10-18	QYFLGILKA	A
CML1	VCFLGILGN	A
CTXN3	FIFLGILIV	A, B
FGF6	LVFLGILVG	A
HERC1	FKFLGILMG	A
IFNG	TLFLGILKN	A
LEMD2	LVFLGILWV	A, B
LIRA4	LLFLGILLF	A
LIRA6	LVFLGILLF	A
MFSD8	SAFLGILNI	A
MGT5B	EKFLGILNK	A
MRGRF	DIFLGILLF	A
MS4A5	VLFLGILIT	A, B
MYH11/14	ASFLGILDI	A
NPTN	WPFLGILAE	A
O10R2	YFFLGILST	A
O56B1	YIFLGILCM	A, B
PAQR1	FLFLGILTM	A, B
SCN7A	SLFLGILAM	A, B, C
SEM4F	GFFLGILAA	A
SMG7	MSFLGILCK	A
TM127	FCFLGILCS	A
XPR1	AGFLGILWC	A
ZN836	LVFLGILPK	A
ZSC18	EQFLGILPD	A
O56B2	YYFLGILAM	A
S35F3	ALFLSILGV	B, C
FCERB	MLFLTILGL	B
GRAM4	FLFLAILRL	B
DB119	YLFLAILLA	B
PCDB5	VMFLAILLL	B
PKDRE	LLFLTILKT	B
I12R1	GSFLSILLV	B
PIGH	AIFITLLGL	C
GPNMB	AIFVTVISL	C
ATP9B	ALFLALVAL	C

SYNG1	ALFLAVLSI	C
FBLN3	ALFLTMLTL	C
UNC5A	ALYVGLIAV	C
S10A2	AVFLALITV	C
CKLF1	AVFLSVVAI	C
CSKI1	AVWLSMIGL	C
TM174	GIFLGLVGI	C
SRTM2	GLFLSLLAI	C
NCKX2	GLFMGLVAI	C
GPR20	GLWLALMAV	C
UNC79	GLYVTLVTL	C
TTMP	SIFLGVITV	C
EFC12	SLFLSLLGL	C
TPSN	SLYLSLVTL	C
DISP1	SMFITLMTM	C
TNF11	SMFVALLGL	C
MYMK	SMWVSLMAL	C
TMM8B	SVWVTVIAM	C
S35A5	TLFLSIVAL	C
HELQ	TLFLSLIGL	C
ZDHC3	TMYIALISL	C
PSMD6	TVYVSMIAL	C

Search A: x-x-F-L-G-I-L-x-x (no filtering according to predicted HLA-A*02:01 IC50)

Search B: x-x-F-L-[SATG]-I-L-x-x, and filtered for predicted HLA-A*02:01 IC50 of 1000 nM or less

Search C: [SATG][VILM][FWY][VILM][SATG][VILM][VILM][SATG][VILM], and filtered for predicted HLA-A*02:01 IC50 of 1000 nM or less

Supplementary Table S4: Published TCR β chain CDR3 sequences of anti-CD20 TCR isolated from humans (Abrahamsen IW et al. Leukemia 2010) compared with the murine TCR CDR3 sequences in this study

Clone	TRB, CDR3 sequences
D1-1	CASSWGQKNIQYF
D1-2	CATAPGLSYEQYF
D1-3	CASSWGRAYEQYF
D1-4	CASSAGVGYEQYF
D1-5	CASSLGSANTQYF
D1-6	CASSPGLTYEQYF
D1-7	CASSQGLTYEQYF
D2-1	CASSWGEGYEQYF
D2-2	CATGGGVSYEQYF
D2-3	CASSLGIAYEQYF
D3-1	CASSHGNGYEQYF
D3-2	CASSETSYEQYF
D3-3	CASSSGLAGVNEQFF
D3-4	CASSLGSLSYEQYF
D3-5	CASTWGPSYEQYF
D3-6	CASSLFGGAGAFF
D3-7	CASSLSDAYEQYF
D4-1	CASSLGNRIGGLFF
D4-2	CASSPGLTYEQYF
D4-3	CASSVGGGYEQYF
D4-4	CASSVAPTGAYGYTF
D4-5	CASSIGYEQFF
D4-6	CASSLGQTYEQYF
D4-7	CASSLGAAYEQYF

Supplementary Table S5: Primer sequences for genotyping of B6.*AireKO* mice.

Target	Prime sequence (5' > 3')
Aire wild type, reverse primer	GGA GAC TTG CCT ATT CCT GTC
Aire mutant, reverse primer	CCG GCG GAT TTG TCC TAC
Forward primer (common)	AGA CTA GGT GTT CCC TCC CAA CCT CAG

Supplementary Table S6: Antibodies and reagents used for flow cytometry analysis

Target antigen	Clone	Conjugate	Company	Catalog #
CD3, mouse	145-2C11	PE-Cy7	BioLegend	100320
CD8a, mouse	53-6.7	FITC	BioLegend	100706
CD8a, mouse	53-6.7	BV421	BioLegend	100738
CD4, mouse	GK1.5	APC-Cy7	BioLegend	100414
TCRβ (TRBC), mouse	H57-597	FITC	eBioscience	4302124
CD19, human	SJ25C1	eFluor 450	eBioscience	48-0198-42
CD22, human	S-HCL-1	APC	BioLegend	363506
CD20, human	2H7	FITC	BioLegend	302304
CD3, human	SK7	PerCP-Cy5.5	BioLegend	344808
CD8a, human	SK1	eFluor 450	eBioscience	48-0087-42
CD4, human	RPA-T4	APC-eFluor 780	eBioscience	47-0049-42
HLA-A2	BB7.2	PE-Cy7	BioLegend	343314
HLA-A2	BB7.2	PE	BioLegend	343306
CD271 (NGFR), human	ME20.4	APC	BioLegend	345108
Streptavidin	N/A	PE	eBioscience	12-4317-87
Viability dye (fixable)	N/A	eFluor 506	eBioscience	65-0866-14
7AAD (BD Via-Probe)	N/A	N/A	BD Biosciences	555815
Flex-T HLA-A*02:01 Monomer UVX	N/A	N/A	BioLegend	280004

Supplementary Table S7: Reagents used for HLA class I-blocking experiment

Target antigen	Clone	Company	Catalog #
HLA-A2	BB7.2	Genscript	U8700CG130
HLA-A,B,C	W6/32	BioLegend	311441
Isotype, mouse IgG2a-kappa	MOPC-173	BioLegend	400281

REFERENCES AND NOTES

1. E. Tran, P. F. Robbins, S. A. Rosenberg, 'Final common pathway' of human cancer immunotherapy: Targeting random somatic mutations. *Nat. Immunol.* **18**, 255–262 (2017).

2. B. Y. Jin, T. E. Campbell, L. M. Draper, S. Stevanović, B. Weissbrich, Z. Yu, N. P. Restifo, S. A. Rosenberg, C. L. Trimble, C. S. Hinrichs, Engineered T cells targeting E7 mediate regression of human papillomavirus cancers in a murine model. *JCI Insight* **3**, e99488 (2018).

3. N. B. Nagarsheth, S. M. Norberg, A. L. Sinkoe, S. Adhikary, T. J. Meyer, J. B. Lack, A. C. Warner, C. Schweitzer, S. L. Doran, S. Korrapati, S. Stevanović, C. L. Trimble, J. A. Kanakry, M. H. Bagheri, E. Ferraro, S. H. Astrow, A. Bot, W. C. Faquin, D. Stroncek, N. Gkitsas, S. Highfill, C. S. Hinrichs, TCR-engineered T cells targeting E7 for patients with metastatic HPV-associated epithelial cancers. *Nat. Med.* **27**, 419–425 (2021).

4. S. Vasileiou, P. D. Lulla, I. Tzannou, A. Watanabe, M. Kuvalekar, W. L. Callejas, M. Bilgi, T. Wang, M. J. Wu, R. Kamble, C. A. Ramos, R. H. Rouce, Z. Zeng, A. P. Gee, B. J. Grilley, J. F. Vera, C. M. Bollard, M. K. Brenner, H. E. Heslop, C. M. Rooney, A. M. Leen, G. Carrum, T-cell therapy for lymphoma using nonengineered multiantigen-targeted T cells is safe and produces durable clinical effects. *J. Clin. Oncol.* **39**, 1415–1425 (2021).

5. N. N. Hunder, H. Wallen, J. Cao, D. W. Hendricks, J. Z. Reilly, R. Rodmyre, A. Jungbluth, S. Gnjatic, J. A. Thompson, C. Yee, Treatment of metastatic melanoma with autologous $CD4^+$ T cells against NY-ESO-1. *N. Engl. J. Med.* **358**, 2698–2703 (2008).

6. P. F. Robbins, R. A. Morgan, S. A. Feldman, J. C. Yang, R. M. Sherry, M. E. Dudley, J. R. Wunderlich, A. V. Nahvi, L. J. Helman, C. L. Mackall, U. S. Kammula, M. S. Hughes, N. P. Restifo, M. Raffeld, C.C. R. Lee, C. L. Levy, Y. F. Li, M. el-Gamil, S. L. Schwarz, C. Laurencot, S. A. Rosenberg, Tumor regression in patients with metastatic synovial cell sarcoma and melanoma using genetically engineered lymphocytes reactive with NY-ESO-1. *J. Clin. Oncol.* **29**, 917–924 (2011).

7. P. F. Robbins, S. H. Kassim, T. L. N. Tran, J. S. Crystal, R. A. Morgan, S. A. Feldman, J. C. Yang, M. E. Dudley, J. R. Wunderlich, R. M. Sherry, U. S. Kammula, M. S. Hughes, N. P.

Restifo, M. Raffeld, C.-C. R. Lee, Y. F. Li, M. el-Gamil, S. A. Rosenberg, A pilot trial using lymphocytes genetically engineered with an NY-ESO-1-reactive T-cell receptor: Long-term follow-up and correlates with response. *Clin. Cancer Res.* **21**, 1019–1027 (2015).

8. A. G. Chapuis, D. N. Egan, M. Bar, T. M. Schmitt, M. S. McAfee, K. G. Paulson, V. Voillet, R. Gottardo, G. B. Ragnarsson, M. Bleakley, C. C. Yeung, P. Muhlhauser, H. N. Nguyen, L. A. Kropp, L. Castelli, F. Wagener, D. Hunter, M. Lindberg, K. Cohen, A. Seese, M. J. McElrath, N. Duerkopp, T. A. Gooley, P. D. Greenberg, T cell receptor gene therapy targeting WT1 prevents acute myeloid leukemia relapse post-transplant. *Nat. Med.* **25**, 1064–1072 (2019).

9. S. Stevanović, A. Pasetto, S. R. Helman, J. J. Gartner, T. D. Prickett, B. Howie, H. S. Robins, P. F. Robbins, C. A. Klebanoff, S. A. Rosenberg, C. S. Hinrichs, Landscape of immunogenic tumor antigens in successful immunotherapy of virally induced epithelial cancer. *Science* **356**, 200–205 (2017).

10. B. J. Cameron, A. B. Gerry, J. Dukes, J. V. Harper, V. Kannan, F. C. Bianchi, F. Grand, J. E. Brewer, M. Gupta, G. Plesa, G. Bossi, A. Vuidepot, A. S. Powlesland, A. Legg, K. J. Adams, A. D. Bennett, N. J. Pumphrey, D. D. Williams, G. Binder-Scholl, I. Kulikovskaya, B. L. Levine, J. L. Riley, A. Varela-Rohena, E. A. Stadtmauer, A. P. Rapoport, G. P. Linette, C. H. June, N. J. Hassan, M. Kalos, B. K. Jakobsen, Identification of a titin-derived HLA-A1-presented peptide as a cross-reactive target for engineered MAGE A3-directed T cells. *Sci. Transl. Med.* **5**, 197ra103 (2013).

11. G. P. Linette, E. A. Stadtmauer, M. V. Maus, A. P. Rapoport, B. L. Levine, L. Emery, L. Litzky, A. Bagg, B. M. Carreno, P. J. Cimino, G. K. Binder-Scholl, D. P. Smethurst, A. B. Gerry, N. J. Pumphrey, A. D. Bennett, J. E. Brewer, J. Dukes, J. Harper, H. K. Tayton-Martin, B. K. Jakobsen, N. J. Hassan, M. Kalos, C. H. June, Cardiovascular toxicity and titin cross-reactivity of affinity-enhanced T cells in myeloma and melanoma. *Blood* **122**, 863–871 (2013).

12. R. A. Morgan, N. Chinnasamy, D. Abate-Daga, A. Gros, P. F. Robbins, Z. Zheng, M. E. Dudley, S. A. Feldman, J. C. Yang, R. M. Sherry, G. Q. Phan, M. S. Hughes, U. S. Kammula,

A. D. Miller, C. J. Hessman, A. A. Stewart, N. P. Restifo, M. M. Quezado, M. Alimchandani, A. Z. Rosenberg, A. Nath, T. Wang, B. Bielekova, S. C. Wuest, N. Akula, F. J. McMahon, S. Wilde, B. Mosetter, D. J. Schendel, C. M. Laurencot, S. A. Rosenberg, Cancer regression and neurological toxicity following anti-MAGE-A3 TCR gene therapy. *J. Immunother.* **36**, 133–151 (2013).

13. L. Jahn, P. Hombrink, R. S. Hagedoorn, M. G. D. Kester, D. M. van der Steen, T. Rodriguez, T. Pentcheva-Hoang, A. H. de Ru, M. P. Schoonakker, M. H. Meeuwsen, M. Griffioen, P. A. van Veelen, J. H. F. Falkenburg, M. H. M. Heemskerk, TCR-based therapy for multiple myeloma and other B-cell malignancies targeting intracellular transcription factor BOB1. *Blood* **129**, 1284–1295 (2017).

14. P. F. Robbins, Y. F. Li, M. el-Gamil, Y. Zhao, J. A. Wargo, Z. Zheng, H. Xu, R. A. Morgan, S. A. Feldman, L. A. Johnson, A. D. Bennett, S. M. Dunn, T. M. Mahon, B. K. Jakobsen, S. A. Rosenberg, Single and dual amino acid substitutions in TCR CDRs can enhance antigen-specific T cell functions. *J. Immunol.* **180**, 6116–6131 (2008).

15. Y. Li, R. Moysey, P. E. Molloy, A.L. Vuidepot, T. Mahon, E. Baston, S. Dunn, N. Liddy, J. Jacob, B. K. Jakobsen, J. M. Boulter, Directed evolution of human T-cell receptors with picomolar affinities by phage display. *Nat. Biotechnol.* **23**, 349–354 (2005).

16. H. M. Bijen, D. M. van der Steen, R. S. Hagedoorn, A. K. Wouters, L. Wooldridge, J. H. F. Falkenburg, M. H.M. Heemskerk, Preclinical strategies to identify off-target toxicity of high-affinity TCRs. *Mol. Ther.* **26**, 1206–1214 (2018).

17. B. Szomolay, J. Liu, P. E. Brown, J. J. Miles, M. Clement, S. Llewellyn-Lacey, G. Dolton, J. Ekeruche-Makinde, A. Lissina, A. J. Schauenburg, A. K. Sewell, S. R. Burrows, M. Roederer, D. A. Price, L. Wooldridge, H. A. Berg, Identification of human viral protein-derived ligands recognized by individual MHCI-restricted T-cell receptors. *Immunol. Cell Biol.* **94**, 573–582 (2016).

18. L. Wooldridge, J. Ekeruche-Makinde, H. A. van den Berg, A. Skowera, J. J. Miles, M. P. Tan, G. Dolton, M. Clement, S. Llewellyn-Lacey, D. A. Price, M. Peakman, A. K. Sewell, A

single autoimmune T cell receptor recognizes more than a million different peptides. *J. Biol. Chem.* **287**, 1168–1177 (2012).

19. M. H. Gee, A. Han, S. M. Lofgren, J. F. Beausang, J. L. Mendoza, M. E. Birnbaum, M. T. Bethune, S. Fischer, X. Yang, R. Gomez-Eerland, D. B. Bingham, L. V. Sibener, R. A. Fernandes, A. Velasco, D. Baltimore, T. N. Schumacher, P. Khatri, S. R. Quake, M. M. Davis, K. C. Garcia, Antigen identification for orphan T cell receptors expressed on tumor-infiltrating lymphocytes. *Cell* **172**, 549–563.e16 (2018).

20. J. J. Adams, S. Narayanan, M. E. Birnbaum, S. S. Sidhu, S. J. Blevins, M. H. Gee, L. V. Sibener, B. M. Baker, D. M. Kranz, K. C. Garcia, Structural interplay between germline interactions and adaptive recognition determines the bandwidth of TCR-peptide-MHC cross-reactivity. *Nat. Immunol.* **17**, 87–94 (2016).

21. M. E. Birnbaum, J. L. Mendoza, D. K. Sethi, S. Dong, J. Glanville, J. Dobbins, E. Özkan, M. M. Davis, K. W. Wucherpfennig, K. C. Garcia, Deconstructing the peptide-MHC specificity of T cell recognition. *Cell* **157**, 1073–1087 (2014).

22. E. de Castro, C. J. A. Sigrist, A. Gattiker, V. Bulliard, P. S. Langendijk-Genevaux, E. Gasteiger, A. Bairoch, N. Hulo, ScanProsite: Detection of PROSITE signature matches and ProRule-associated functional and structural residues in proteins. *Nucleic Acids Res.* **34**(Web Server issue), W362–W365 (2006).

23. A. B. Riemer, D. B. Keskin, G. Zhang, M. Handley, K. S. Anderson, V. Brusic, B. Reinhold, E. L. Reinherz, A conserved E7-derived cytotoxic T lymphocyte epitope expressed on human papillomavirus 16-transformed HLA-A2^{+} epithelial cancers. *J. Biol. Chem.* **285**, 29608–29622 (2010).

24. M. Harndahl, M. Rasmussen, G. Roder, S. Buus, Real-time, high-throughput measurements of peptide-MHC-I dissociation using a scintillation proximity assay. *J. Immunol. Methods* **374**, 5–12 (2011).

25. S. Henikoff, J. G. Henikoff, Amino acid substitution matrices from protein blocks. *Proc. Natl. Acad. Sci. U.S.A.* **89**, 10915–10919 (1992).

26. C. Pommie, S. Levadoux, R. Sabatier, G. Lefranc, M.-P. Lefranc, IMGT standardized criteria for statistical analysis of immunoglobulin V-REGION amino acid properties. *J. Mol. Recognit.* **17**, 17–32 (2004).

27. D. Bordo, P. Argos, Suggestions for “safe” residue substitutions in site-directed mutagenesis. *J. Mol. Biol.* **217**, 721–729 (1991).

28. D. J. Barlow, J. M. Thornton, Helix geometry in proteins. *J. Mol. Biol.* **201**, 601–619 (1988).

29. M. Uhlen, L. Fagerberg, B. M. Hallström, C. Lindskog, P. Oksvold, A. Mardinoglu, Å. Sivertsson, C. Kampf, E. Sjöstedt, A. Asplund, I. M. Olsson, K. Edlund, E. Lundberg, S. Navani, C. Al-Khalili Szigyarto, J. Odeberg, D. Djureinovic, J. O. Takanen, S. Hober, T. Alm, P.-H. Edqvist, H. Berling, H. Tegel, J. Mulder, J. Rockberg, P. Nilsson, J. M. Schwenk, M. Hamsten, K. von Feilitzen, M. Forsberg, L. Persson, F. Johansson, M. Zwahlen, G. von Heijne, J. Nielsen, F. Pontén, Tissue-based map of the human proteome. *Science* **347**, 1260419 (2015).

30. P. J. Thul, L. Åkesson, M. Wiking, D. Mahdessian, A. Geladaki, H. Ait Blal, T. Alm, A. Asplund, L. Björk, L. M. Breckels, A. Bäckström, F. Danielsson, L. Fagerberg, J. Fall, L. Gatto, C. Gnann, S. Hober, M. Hjelmare, F. Johansson, S. Lee, C. Lindskog, J. Mulder, C. M. Mulvey, P. Nilsson, P. Oksvold, J. Rockberg, R. Schutten, J. M. Schwenk, Å. Sivertsson, E. Sjöstedt, M. Skogs, C. Stadler, D. P. Sullivan, H. Tegel, C. Winsnes, C. Zhang, M. Zwahlen, A. Mardinoglu, F. Pontén, K. von Feilitzen, K. S. Lilley, M. Uhlén, E. Lundberg, A subcellular map of the human proteome. *Science* **356**, eaal3321 (2017).

31. E. J. Wherry, M. Kurachi, Molecular and cellular insights into T cell exhaustion. *Nat. Rev. Immunol.* **15**, 486–499 (2015).

32. M. A. ElTanbouly, R. J. Noelle, Rethinking peripheral T cell tolerance: Checkpoints across a T cell's journey. *Nat. Rev. Immunol.* **21**, 257–267 (2021).

33. W. R. Pearson, Selecting the right similarity-scoring matrix. *Curr. Protoc. Bioinformatics* **43**, 3.5.1–3.5.9 (2013).

34. J. Ishizuka, K. Grebe, E. Shenderov, B. Peters, Q. Chen, Y.C. Peng, L. Wang, T. Dong, V. Pasquetto, C. Oseroff, J. Sidney, H. Hickman, V. Cerundolo, A. Sette, J. R. Bennink, A. McMichael, J. W. Yewdell, Quantitating T cell cross-reactivity for unrelated peptide antigens. *J. Immunol.* **183**, 4337–4345 (2009).

35. L. Wooldridge, B. Laugel, J. Ekeruche, M. Clement, H. A. van den Berg, D. A. Price, A. K. Sewell, CD8 controls T cell cross-reactivity. *J. Immunol.* **185**, 4625–4632 (2010).

36. S. H. Chiou, D. Tseng, A. Reuben, V. Mallajosyula, I. S. Molina, S. Conley, J. Wilhelmy, A. M. McSween, X. Yang, D. Nishimiya, R. Sinha, B. Y. Nabet, C. Wang, J. B. Shrager, M. F. Berry, L. Backhus, N. S. Lui, H. A. Wakelee, J. W. Neal, S. K. Padda, G. J. Berry, A. Delaidelli, P. H. Sorensen, E. Sotillo, P. Tran, J. A. Benson, R. Richards, L. Labanieh, D. D. Klysz, D. M. Louis, S. A. Feldman, M. Diehn, I. L. Weissman, J. Zhang, I. I. Wistuba, P. A. Futreal, J. V. Heymach, K. C. Garcia, C. L. Mackall, M. M. Davis, Global analysis of shared T cell specificities in human non-small cell lung cancer enables HLA inference and antigen discovery. *Immunity* **54**, 586–602.e8 (2021).

37. A. D. Martin, X. Wang, M. L. Sandberg, K. R. Negri, M. L. Wu, D. Toledo Warshaviak, G. B. Gabrelow, M. E. McElvain, B. Lee, M. E. Daris, H. Xu, A. Kamb, Re-examination of MAGE-A3 as a T-cell therapeutic target. *J. Immunother.* **44**, 95–105 (2021).

38. K. C. Garcia, J. J. Adams, D. Feng, L. K. Ely, The molecular basis of TCR germline bias for MHC is surprisingly simple. *Nat. Immunol.* **10**, 143–147 (2009).

39. A. K. Sewell, Why must T cells be cross-reactive? *Nat. Rev. Immunol.* **12**, 669–677 (2012).

40. T. P. Arstila, A. Casrouge, V́.. Baron, J. Even, J. Kanellopoulos, P. Kourilsky, A direct estimate of the human αβ T cell receptor diversity. *Science* **286**, 958–961 (1999).

41. J. Ekeruche-Makinde, J. J. Miles, H. A. van den Berg, A. Skowera, D. K. Cole, G. Dolton, A. J. A. Schauenburg, M. P. Tan, J. M. Pentier, S. Llewellyn-Lacey, K. M. Miles, A. M. Bulek,

M. Clement, T. Williams, A. Trimby, M. Bailey, P. Rizkallah, J. Rossjohn, M. Peakman, D. A. Price, S. R. Burrows, A. K. Sewell, L. Wooldridge, Peptide length determines the outcome of TCR/peptide-MHCI engagement. *Blood* **121**, 1112–1123 (2013).

42. E. C. Border, J. P. Sanderson, T. Weissensteiner, A. B. Gerry, N. J. Pumphrey, Affinity-enhanced T-cell receptors for adoptive T-cell therapy targeting MAGE-A10: Strategy for selection of an optimal candidate. *Onco. Targets Ther.* **8**, e1532759 (2019).

43. A. K. Bentzen, L. Such, K. K. Jensen, A. M. Marquard, L. E. Jessen, N. J. Miller, C. D. Church, R. Lyngaa, D. M. Koelle, J. C. Becker, C. Linnemann, T. N. M. Schumacher, P. Marcatili, P. Nghiem, M. Nielsen, S. R. Hadrup, T cell receptor fingerprinting enables in-depth characterization of the interactions governing recognition of peptide-MHC complexes. *Nat. Biotechnol.* **36**, 1191–1196 (2018).

44. L. Cai, L. D. Caraballo Galva, Y. Peng, X. Luo, W. Zhu, Y. Yao, Y. Ji, Y. He, Preclinical studies of the off-target reactivity of AFP_{158}-specific TCR engineered T cells. *Front. Immunol.* **11**, 607 (2020).

45. X. Luo, H. Cui, L. Cai, W. Zhu, W.-C. Yang, M. Patrick, S. Zhu, J. Huang, X. Yao, Y. Yao, Y. He, Y. Ji, Selection of a clinical lead TCR targeting α-fetoprotein-positive liver cancer based on a balance of risk and benefit. *Front. Immunol.* **11**, 623 (2020).

46. X. Pan, L.C. Huang, T. Dong, Y. Peng, V. Cerundolo, S. McGowan, G. Ogg, Combinatorial HLA-peptide bead libraries for high throughput identification of $CD8^+$ T cell specificity. *J. Immunol. Methods* **403**, 72–78 (2014).

47. A. V. Joglekar, M. T. Leonard, J. D. Jeppson, M. Swift, G. Li, S. Wong, S. Peng, J. M. Zaretsky, J. R. Heath, A. Ribas, M. T. Bethune, D. Baltimore, T cell antigen discovery via signaling and antigen-presenting bifunctional receptors. *Nat. Methods* **16**, 191–198 (2019).

48. J. Glanville, H. Huang, A. Nau, O. Hatton, L. E. Wagar, F. Rubelt, X. Ji, A. Han, S. M. Krams, C. Pettus, N. Haas, C. S. L. Arlehamn, A. Sette, S. D. Boyd, T. J. Scriba, O. M.

Martinez, M. M. Davis, Identifying specificity groups in the T cell receptor repertoire. *Nature* **547**, 94–98 (2017).

49. H. Huang, C. Wang, F. Rubelt, T. J. Scriba, M. M. Davis, Analyzing the *Mycobacterium tuberculosis* immune response by T-cell receptor clustering with GLIPH2 and genome-wide antigen screening. *Nat. Biotechnol.* **38**, 1194–1202 (2020).

50. M. N. Duong, E. Erdes, M. Hebeisen, N. Rufer, Chronic TCR-MHC (self)-interactions limit the functional potential of TCR affinity-increased CD8 T lymphocytes. *J. Immunother. Cancer* **7**, 284 (2019).

51. B. Laugel, H. A. van den Berg, E. Gostick, D. K. Cole, L. Wooldridge, J. Boulter, A. Milicic, D. A. Price, A. K. Sewell, Different T cell receptor affinity thresholds and CD8 coreceptor dependence govern cytotoxic T lymphocyte activation and tetramer binding properties. *J. Biol. Chem.* **282**, 23799–23810 (2007).

52. J. Bae, J. A. Martinson, H. G. Klingemann, Identification of CD19 and CD20 peptides for induction of antigen-specific CTLs against B-cell malignancies. *Clin. Cancer Res.* **11**, 1629–1638 (2005).

53. L. Jahn, D. M. van der Steen, R. S. Hagedoorn, P. Hombrink, M. G. D. Kester, M. P. Schoonakker, D. de Ridder, P. A. van Veelen, J. H. F. Falkenburg, M. H. M. Heemskerk, Generation of CD20-specific TCRs for TCR gene therapy of $CD20^{low}$ B-cell malignancies insusceptible to CD20-targeting antibodies. *Oncotarget* **7**, 77021–77037 (2016).

54. L. Jahn, R. S. Hagedoorn, D. M. van der Steen, P. Hombrink, M. G. D. Kester, M. P. Schoonakker, D. de Ridder, P. A. van Veelen, J. H. F. Falkenburg, M. H. M. Heemskerk, A CD22-reactive TCR from the T-cell allorepertoire for the treatment of acute lymphoblastic leukemia by TCR gene transfer. *Oncotarget* **7**, 71536–71547 (2016).

55. I. W. Abrahamsen, E. Stronen, S. Wälchli, J. N. Johansen, S. Kjellevoll, S. Kumari, M. Komada, G. Gaudernack, G. Tjonnfjord, M. Toebes, T. N. Schumacher, F. Lund-Johansen, J.

Olweus, Targeting B cell leukemia with highly specific allogeneic T cells with a public recognition motif. *Leukemia* **24**, 1901–1909 (2010).

56. N. Mensali, F. Ying, V. O. Y. Sheng, W. Yang, E. Walseng, S. Kumari, L.-E. Fallang, A. Kolstad, W. Uckert, K. J. Malmberg, S. Wälchli, J. Olweus, Targeting B-cell neoplasia with T-cell receptors recognizing a CD20-derived peptide on patient-specific HLA. *Oncoimmunology* **5**, e1138199 (2016).

57. A. L. Amir, L. J. A. D'Orsogna, D. L. Roelen, M. M. van Loenen, R. S. Hagedoorn, R. de Boer, M. A. W. G. van der Hoorn, M. G. D. Kester, I. I. N. Doxiadis, J. H. F. Falkenburg, F. H. J. Claas, M. H. M. Heemskerk, Allo-HLA reactivity of virus-specific memory T cells is common. *Blood* **115**, 3146–3157 (2010).

58. A. Haga-Friedman, M. Horovitz-Fried, C. J. Cohen, Incorporation of transmembrane hydrophobic mutations in the TCR enhance its surface expression and T cell functional avidity. *J. Immunol.* **188**, 5538–5546 (2012).

59. C. J. Cohen, Y. F. Li, M. el-Gamil, P. F. Robbins, S. A. Rosenberg, R. A. Morgan, Enhanced antitumor activity of T cells engineered to express T-cell receptors with a second disulfide bond. *Cancer Res.* **67**, 3898–3903 (2007).

60. Y. Kim, J. Ponomarenko, Z. Zhu, D. Tamang, P. Wang, J. Greenbaum, C. Lundegaard, A. Sette, O. Lund, P. E. Bourne, M. Nielsen, B. Peters, Immune epitope database analysis resource. *Nucleic Acids Res.* **40**(Web Server issue), W525–W530 (2012).

61. M. O. S. Dayhoff, R. M. Schwartz, B. C. Orcutt, A model of evolutionary change in proteins, in *Atlas of Protein Sequence and Structure* (National Biomedical Research Foundation, 1978).

62. B. Rodenko, M. Toebes, S. R. Hadrup, W. J. E. van Esch, A. M. Molenaar, T. N. M. Schumacher, H. Ovaa, Generation of peptide-MHC class I complexes through UV-mediated ligand exchange. *Nat. Protoc.* **1**, 1120–1132 (2006).

63. M. S. Anderson, E. S. Venanzi, L. Klein, Z. Chen, S. P. Berzins, S. J. Turley, H. von Boehmer, R. Bronson, Á.. Dierich, C. Benoist, D. Mathis, Projection of an immunological self shadow within the thymus by the aire protein. *Science* **298**, 1395–1401 (2002).

64. O. Wagih, ggseqlogo: A versatile R package for drawing sequence logos. *Bioinformatics* **33**, 3645–3647 (2017).

65. M. Nielsen, C. Lundegaard, P. Worning, S. L. Lauemøller, K. Lamberth, S. Buus, S. Brunak, O. Lund, Reliable prediction of T-cell epitopes using neural networks with novel sequence representations. *Protein Sci.* **12**, 1007–1017 (2003).

66. C. Lundegaard, K. Lamberth, M. Harndahl, S. Buus, O. Lund, M. Nielsen, NetMHC-3.0: Accurate web accessible predictions of human, mouse and monkey MHC class I affinities for peptides of length 8–11. *Nucleic Acids Res.* **36**(Web Server issue), W509–W512 (2008).

67. M. Andreatta, M. Nielsen, Gapped sequence alignment using artificial neural networks: Application to the MHC class I system. *Bioinformatics* **32**, 511–517 (2016).

68. B. Peters, A. Sette, Generating quantitative models describing the sequence specificity of biological processes with the stabilized matrix method. *BMC Bioinformatics* **6**, 132 (2005).

69. J. Sidney, E. Assarsson, C. Moore, S. Ngo, C. Pinilla, A. Sette, B. Peters, Quantitative peptide binding motifs for 19 human and mouse MHC class I molecules derived using positional scanning combinatorial peptide libraries. *Immunome Res.* **4**, 2 (2008).

70. E. W. Sayers, E. E. Bolton, J. R. Brister, K. Canese, J. Chan, D. C. Comeau, R. Connor, K. Funk, C. Kelly, S. Kim, T. Madej, A. Marchler-Bauer, C. Lanczycki, S. Lathrop, Z. Lu, F. Thibaud-Nissen, T. Murphy, L. Phan, Y. Skripchenko, T. Tse, J. Wang, R. Williams, B. W. Trawick, K. D. Pruitt, S. T. Sherry, Database resources of the national center for biotechnology information. *Nucleic Acids Res.* **50**(D1), D20–D26 (2022).

71. S. F. Altschul, W. Gish, W. Miller, E. W. Myers, D. J. Lipman, Basic local alignment search tool. *J. Mol. Biol.* **215**, 403–410 (1990).
